# Biogenesis, preparation, characterization, therapeutic mechanisms and safety evaluation of plant-derived exosome-like nanovesicles in the treatment of ulcerative colitis

**DOI:** 10.1186/s12951-026-04209-4

**Published:** 2026-03-21

**Authors:** Cailan Li, Luyou Zhao, Xuefei Wang, Jingjing Wu, Haohui Chen, Qiang Lu

**Affiliations:** 1 Department of Pharmacology, Zunyi Medical University, Zhuhai Campus, Zhuhai, 519041 PR China; 2 Key Laboratory of Basic Pharmacology of Ministry of Education and Joint International Research Laboratory of Ethnomedicine of Ministry of Education, Zunyi Medical University, Zunyi, 563000 PR China; 3 Key Laboratory of Basic Pharmacology of Guizhou Province and School of Pharmacy, Zunyi Medical University, Zunyi, 563000 PR China; 4 Department of Pharmaceutical Sciences, Zunyi Medical University, Zhuhai Campus, Zhuhai, 519041 PR China

**Keywords:** Plant-derived exosome-like nanovesicles, Colitis, Therapeutic effect, Molecular mechanism, Active components, Safety

## Abstract

Ulcerative colitis remains a challenging clinical condition due to its complex etiology and the limitations of current therapies. Plant-derived exosome-like nanovesicles (PDELNs) represent a new class of natural nanotherapeutics with significant potential for ulcerative colitis treatment. These nanoparticles exhibit high biocompatibility, the ability to cross biological barriers, and carry a rich cargo of bioactive molecules. This review synthesizes progress in PDELN research over the past decade, focusing on established preparation methods and their multi-targeted therapeutic mechanisms. The current isolation methods of the anti-colitis PDELNs are mainly various types of centrifugation, including ultracentrifugation, differential centrifugation, density gradient centrifugation and their combination. Therapeutically, PDELNs alleviate colitis through potent anti-inflammatory effects, gut microbiota remodeling, and immune response regulation. These effects are attributed to key bioactive components such as plant microRNAs, metabolites, and lipids. Notably, PDELNs demonstrate an excellent safety profile without reported toxicity. Their natural origin, multi-targeted mechanisms, and favorable biosafety profile make PDELNs a promising next-generation therapeutic candidate against colitis, effectively bridging traditional phytotherapy and modern nanomedicine.

**Graphical Abstract:**

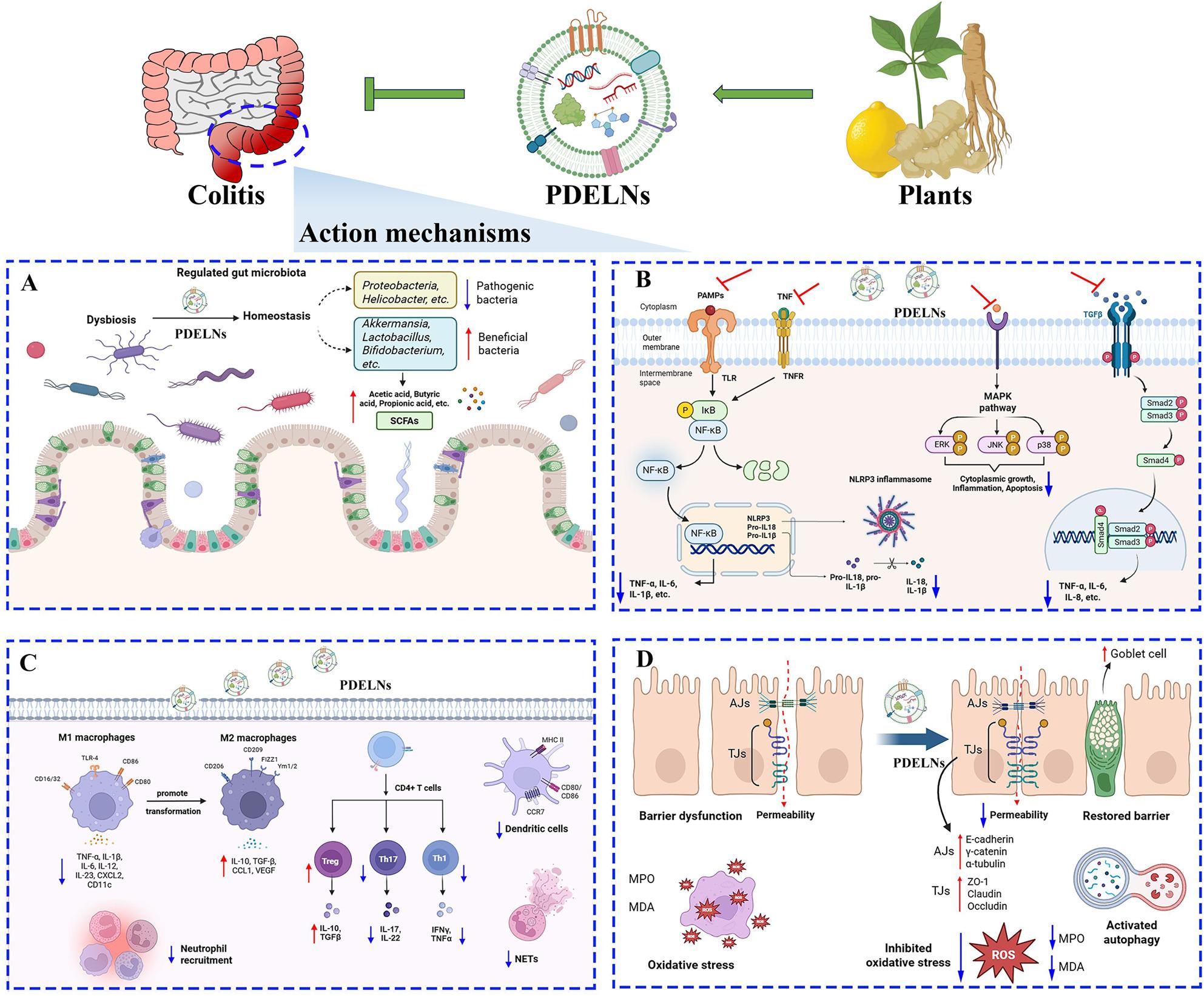

## Introduction

Ulcerative colitis is a chronic inflammatory bowel disease primarily affecting the rectum and colon [1]. Colitis, characterized by its difficulty in treatment, high recurrence rate, and potential for malignant transformation, severely impacts patients’ quality of life and imposes a significant economic burden on society [2]. It has been listed by the World Health Organization (WHO) as one of the modern refractory diseases [3]. The disease demonstrates increasing incidence globally, particularly affecting adults aged 20–50 years without significant gender predominance [4, 5]. While colitis itself has low mortality, long-standing disease significantly increases colorectal cancer risk [6]. The etiology of colitis remains incompletely understood, and its pathogenesis is closely associated with a complex interplay of genetic, environmental, microbial, and immune factors [7]. Modern medicine has yet to develop curative therapies or pharmaceutical agents for colitis [8].

The therapeutic objectives for colitis encompass controlling inflammation, alleviating symptoms, preventing and managing complications, and improving patients’ quality of life [9]. Common therapeutic approaches for colitis comprise supportive management, pharmacotherapy, and surgical intervention [10]. Supportive management primarily involves adequate rest, psychological adjustment, and nutritional interventions, among other foundational measures [11]. Commonly used drugs in colitis clinical practice include 5-aminosalicylates, glucorticosteroids, immunomodulators, etc [12]. However, these pharmacological agents are constrained by limited efficacy, high relapse rates, susceptibility to loss of response, and significant adverse effects, highlighting an urgent imperative to develop safe and effective next-generation therapeutic approaches [13].

In recent years, the emerging treatment methods based on extracellular vesicles (EVs) are gradually gaining attention in the field of colitis treatment [14]. EVs are a collective term for various types of vesicles secreted by cells. They possess membrane structures and can act as information carriers, participating in intercellular communication [15]. EVs are mainly divided into three types: exosomes, microvesicles, and apoptotic bodies [16]. Exosomes are membranous vesicles with a diameter of 30–150 nm, released by the fusion of multivesicular bodies and cell membranes, featuring a lipid bilayer structure and containing components such as lipids, proteins, nucleic acids, and small molecule compounds [17, 18].

Previous studies on exosomes have mainly focused on those derived from animal cells. It is only in the past decade that plant exosomes, commonly referred to as plant-derived exosome-like nanovesicles/nanoparticles (PDELNs), have gradually attracted the attention of researchers. Their sizes are generally between 30 and 400 nm [19]. PDELNs have attracted widespread attention due to their wide sources, high yields, and significant activity [20]. Studies conducted on PDELNs derived from plants such as ginseng, ginger, turmeric, and garlic have demonstrated broad pharmacological activities in areas including tumors, metabolic diseases, and inflammatory disorders [21].

Compared to conventional therapeutic strategies, PDELNs present distinct advantages for colitis treatment. As naturally derived nanovesicles, they exhibit high biocompatibility and low toxicity, potentially overcoming the safety concerns associated with long-term use of immunomodulators and biologics [22]. Moreover, their multi-component nature enables them to simultaneously target multiple pathological processes in colitis, offering a potential solution to the limited efficacy and drug resistance encountered with single-target agents [23]. Additionally, their nanoscale structure and lipid bilayer composition facilitate enhanced stability and targeted delivery to inflamed intestinal tissues, improving the bioavailability of active constituents and addressing the challenge of low therapeutic agent accumulation at disease sites [24]. As part of the current advancements in medicine and nanotechnology, PDELNs may open up new avenues for drug discovery and application, with the potential to develop into next-generation therapeutic agents [25]. Based on current evidence, PDELNs derived from multiple plants have demonstrated considerable therapeutic potential for treating colitis, attracting significant scientific attention [26].

This study comprehensively summarizes the biogenesis, morphology and composition, preparation methods, characterization techniques, the efficacy and underlying mechanisms, as well as the safety of PDELNs in treating colitis. We further discuss the challenges and opportunities in this field, aiming for this work to provide direction and theoretical guidance for novel drug development targeting colitis.

## Biogenesis of PDELNs

**Fig. 1:**
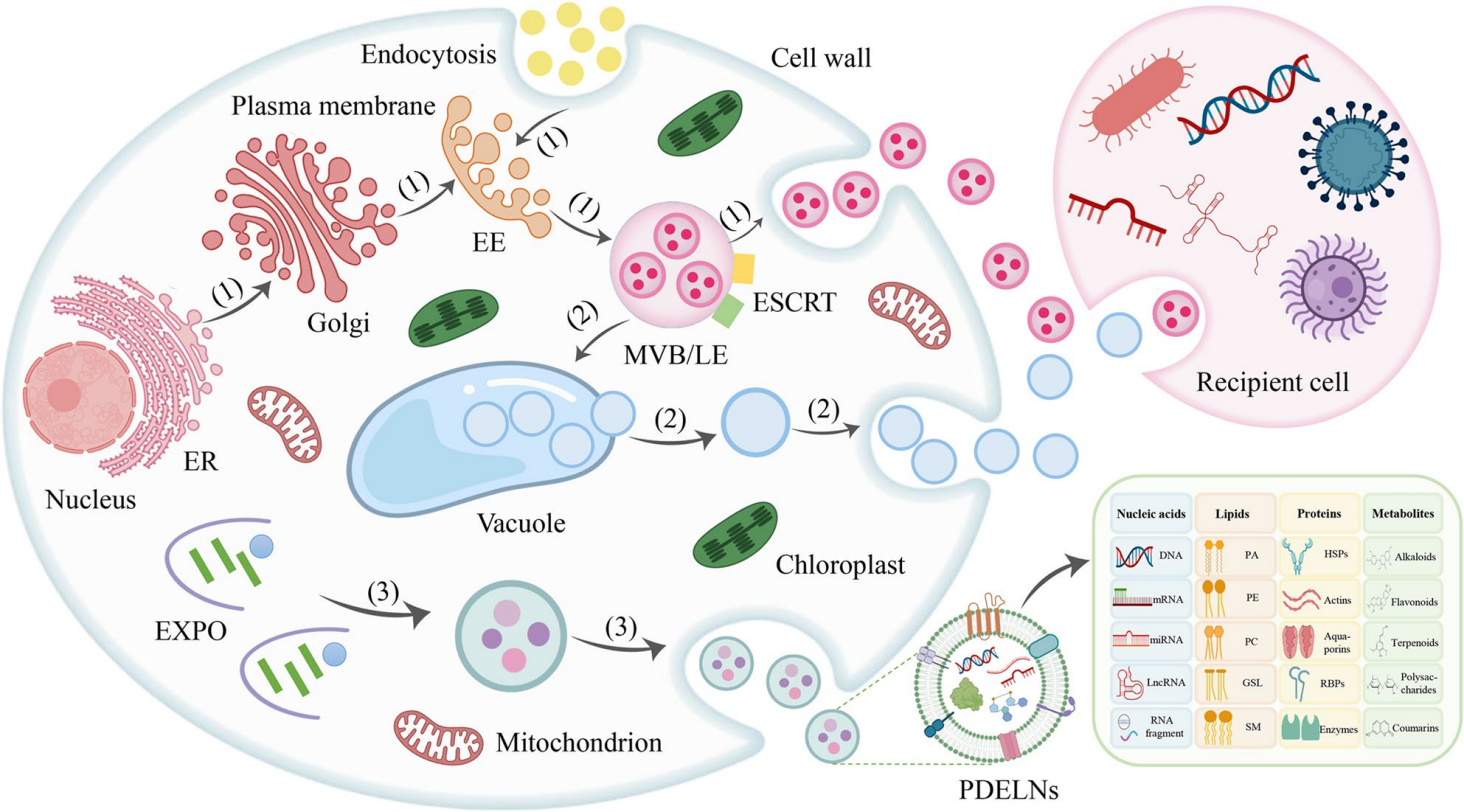
Biogenesis and secretion of exosome-like nanovesicles of plant origin. (1) MVB pathway; (2) vacuole pathway; (3) EXPO pathway. ER, endoplasmic reticulum; EE, early endosome; MVB, multivesicular body; LE, lately endosome; ESCRT, endosomal sorting complex required for transport; EXPO, exocyst-positive organelles; PDELNs, plant-derived exosome-like nanovesicles

The plant-derived exosome-like nanovesicles (PDELNs) are a kind of membranous vesicle that are naturally secreted by plant cells and have morphological characteristics and biological functions resembling those of mammalian exosomes [27]. Current researches have identified three pathways (Fig. 1) for the biogenesis and secretion of PDELNs: the multivesicular body (MVB) pathway, the exocyst-positive organelle (EXPO) pathway, and the vacuole pathway [28]. Among them, MVB is the main biogenesis pathway of plant exosomes [29]. This process starts with the inward depression of the cytoplasmic membrane to form an early endosome. Early endosomes mature further to form late endosomes. It also makes connections with organelles such as the trans-Golgi network. This is followed by further invagination of late endosomes to form MVB. The inner lumen of MVB shapes intraluminal vesicles (ILVs), which eventually fuse with the cellular membrane to release exosomes [30]. MVB formation is modulated by various molecular mechanisms involving the ESCRT complex (0, I, II, III), SNARE proteins, Rab GTPase, and others [31]. Among them, the ESCRT complex plays a significant role in the formation of MVBs and the wrapping up of ILVs and is also involved in plasma membrane budding and division. Rab GTPase, on the other hand, regulates membrane fusion and MVB transport to ensure correct secretion of exosomes. Moreover, a family of four transmembrane proteins, including CD63, CD9, CD82, and CD81, is also implicated in MVB production and exosome secretion [32].

PDELNs can additionally originate in the EXPO pathway. This pathway is a non-dependent pathway and is a secretory pathway specific to plant cells [33]. The double-membrane structure of the EXPO releases a monolayer of vesicles into the extra-cellular environment after fusion with the plasma membrane [34]. EXPO formation is intimately associated with the outer vesicle protein complex, most especially the EXO70E2 subunit, which is crucial to the positioning and functioning of EXPO [35]. An additional PDELNs biogenesis pathway involves the vacuole pathway. Vacuoles, among the plant cells’ power organelles, are critical to the production of PDELNs. Vacuoles contain hydrolytic enzymes and defense proteins. Upon plant infestation by a pathogen, they can fuse with the plasma membrane and release defense materials outside the cellular to suppress pathogen proliferation [36]. In the vacuole pathway, MVB first releases its contents (ILVs) into the vacuoles. Afterwards, it fuses with the plasma membrane and releases the vacuoles into the extra-cellular environment [37]. Despite the current understanding of the biogenesis pathways of PDELNs, the regulation of their mechanisms, as well as the synergistic effects between multiple pathways, still need to be further explored.

### Preparation methods of PDELNs

**Fig. 2:**
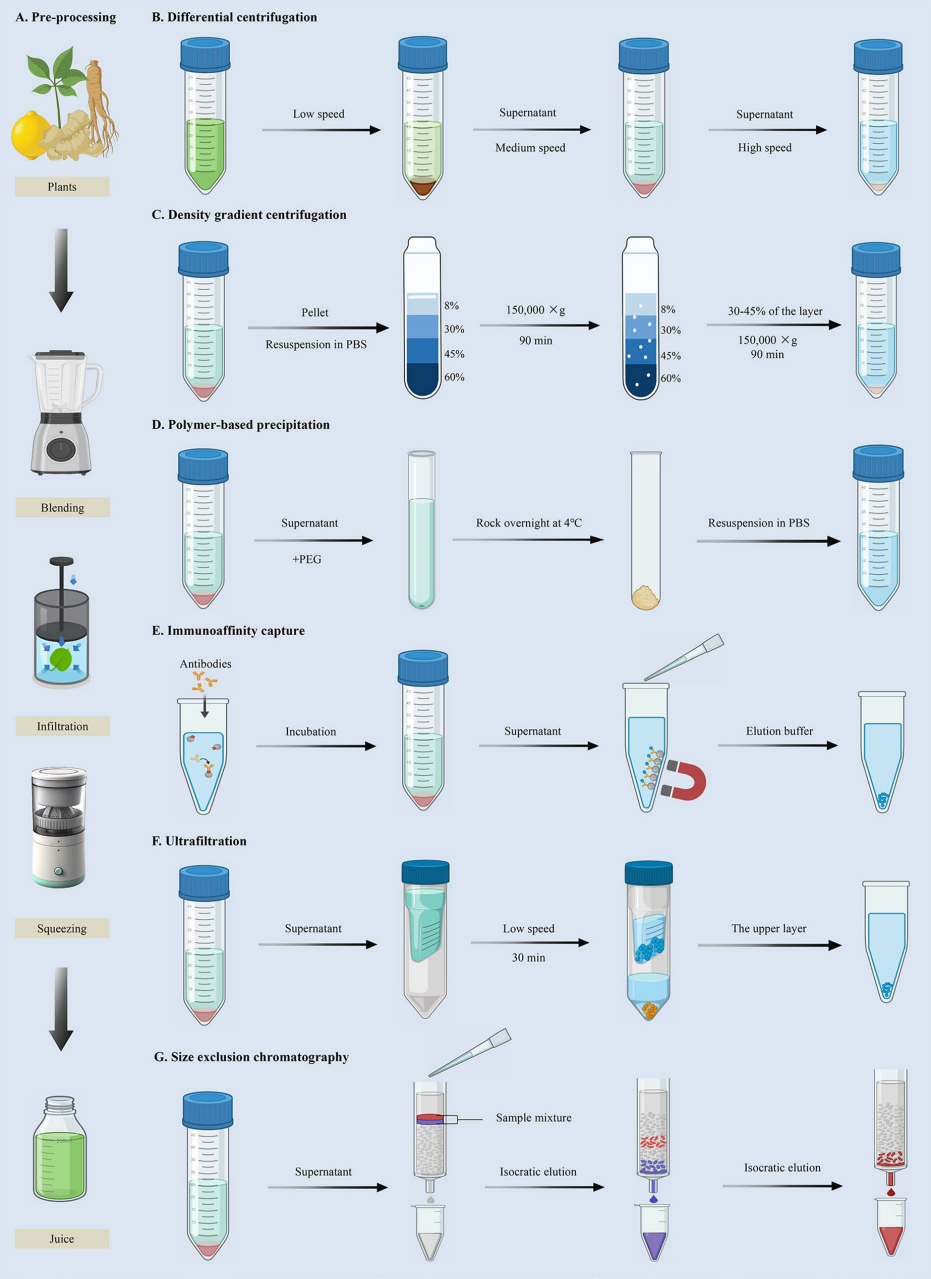
The main preparation methods of plant-derived exosome-like nanovesicles (PDELNs). (**A**) Pre-processing of plant organs or tissues, such as blending, infiltration and squeezing. The subsequent phase employs the following techniques including (**B**) differential centrifugation (DC), (**C**) density gradient centrifugation (DGC), (**D**) polymer-based precipitation (PBP), (**E**) immunoaffinity capture (IC), (**F**) ultrafiltration (UF), and (**G**) size exclusion chromatography (SEC) to obtain pure PDELNs

The separation methods (Fig. 2) of PDELNs mainly include differential centrifugation (DC), density gradient centrifugation (DGC), ultrafiltration (UF), size exclusion chromatography (SEC), polymer-based precipitation (PBP), and immunoaffinity capture (IC) [38]. Currently, the main preparation method of PDELNs is DC combined with DGC. Compared to other methods, this method can separate PDELNs with high purity and good integrity, making it more suitable for large-scale production [39].

## Pre-processing of plant tissues

The roots, stems, leaves, flowers, bark, fruits, and seeds of plants can all be used to extract PDELNs. For fresh plant tissues, blending is the main pre-processing method, such as ginger-derived PDELNs, garlic-derived PDELNs, tea-derived PDELNs, etc [40]. The blending method for pre-processing plant tissues is easy to operate, efficient, and high-yielding, but it can cause cell membrane rupture, release of cellular contents, and rearrangement of membrane structure [41]. Next is the vacuum infiltration method, which obtains cells with intact structures and less contamination of cellular contents, but the operation is relatively cumbersome, time-consuming, and low in yield [42]. Some researches have compared the blending method with the vacuum infiltration method and found that PDELNs obtained by the vacuum infiltration method have smaller particle size, lower zeta potential, lower density, and higher lipid membrane thickness [43].

There are a wide variety of plant species, and the extraction of PDELNs from most fresh plant tissues can be pre-processed by blending. Fresh plant tissues containing more juice can be directly squeezed or juiced [44]. Some plant tissues rich in juice have a high fiber content, and juice extraction produces a large amount of flocculent fiber complexes that are difficult to remove during centrifugation/filtration [45]. Squeezing is a more suitable method for collecting juice. Dried or low juice plant tissues can be juiced by adding an appropriate amount of pre-cooled phosphate buffer solution [46]. For fresh leaves, PDELNs can be obtained by collecting apoplastic washing fluid through infiltration. Plant tissues with high pectin content, such as fruits and seeds, are not suitable for juice extraction [47]. It is recommended to use enzymatic digestion of cell walls for pretreatment.

## Differential centrifugation

DC refers to centrifuging the pre-treated juice at different speeds for different durations. Normally, the pre-treated juice first undergoes low-speed centrifugation (500–10,000 ×g) to remove plant fibers, large particles, dead cells and cellular debris, followed by ultracentrifugation (40,000–100,000 ×g) to obtain PDELNs [48]. The entire process is carried out at 4 ℃. Different plant tissues require different centrifugation speeds and times. This method is simple to operate, but requires a large amount of sample, expensive instruments, and a long processing time [49]. Meanwhile, it is prone to contamination by riboproteins and agglomerations. Moreover, prolonged centrifugation may damage the structure of PDELNs, affecting subsequent experiments, especially functional analysis [50].

## Density gradient centrifugation

DGC refers to the method of distinguishing PDELNs by causing different components with different sedimentation coefficients to settle at a certain speed under ultracentrifugal force, forming bands in different density gradient regions [51]. Commonly used inert substances for gradient construction are sucrose, iodixanol, and others. Therefore, DGC mainly includes sucrose density gradient centrifugation (SDGC) and iodixanol density gradient centrifugation (IDGC) [52]. PDELNs isolated by this method exhibit high purity, but the procedure is labor-intensive, time-consuming, and highly sensitive to centrifugation duration. Moreover, it cannot exclude nanoparticles with densities similar to PDELNs [53].

## Ultrafiltration

UF refers to the use of membrane filters with specific pore sizes to selectively separate PDELNs from other components in juice [54]. This method can greatly save time, improve efficiency, does not require special equipment, and does not affect the biological activity of PDELNs due to operational issues [55]. It is often used as an alternative to DC and DGC. Similarly, UF also has certain drawbacks. The biggest problem faced is membrane blockage, which can shorten the service life of some expensive filter membranes and lead to a decrease in efficiency [56]. Tangential flow filtration can partially mitigate membrane clogging caused by the accumulation of large particles on the filter surface. Additionally, excessive pressure during UF may cause mechanical damage to PDELNs, potentially compromising downstream experiments [57].

### Size exclusion chromatography

SEC is a method whereby the components of plant juice can be separated based on different sizes when passing through a fixed phase composed of porous resin particles [58]. Particles of different sizes in plant juice exhibit varying retention times, which facilitates size-based separation. SEC can retain the natural biological activity of isolated PDELNs. Unlike centrifugation and ultrafiltration, SEC does not affect the structure and integrity of PDELNs through passive gravity flow [59]. The natural state of PDELNs can be better preserved by using elution buffers with physiological osmolarity and viscosity, such as phosphate-buffered saline. However, impurities of similar size to PDELNs will be eluted together with the eluent [60]. Moreover, the challenge of its large-scale industrialization lies in relatively high equipment costs and the need for a significant amount of time and additional enrichment methods [61].

## Polymer-Based precipitation

Highly hydrophilic polymers interact with water molecules around PDELNs, creating a hydrophobic microenvironment that causes them to precipitate [62]. Among various hydrophilic polymers, polyethylene glycol (PEG) is a non-toxic polymer with excellent biocompatibility, serving as a commonly used excipient in pharmaceutical products [63]. It also possesses the ability to reshape the water solubility of surrounding materials and has been widely utilized. The application of PEG precipitation for isolating PDELNs shows great potential as a scalable separation technique [64]. The advantages of PBP lie in low equipment requirements, high sample compatibility, and high efficiency. However, it requires a long processing time and complex compound removal steps, which can affect downstream analysis and quantitative results. PDELNs prepared by PBP exhibit lower purity compared to other approaches, but can be further optimized [65].

## Immunoaffinity capture

Researchers have immobilized antibodies on solid surfaces for PDELNs isolation, successfully developing the first immunomagnetic beads targeting the membrane proteins of PDELNs [66]. Due to its large surface area and nearly uniform process, this method can achieve high capture efficiency and sensitivity. Additionally, it can accommodate a larger initial sample volume, thereby allowing for scaling up or down for specific applications [67]. However, it should be considered that the non-neutral pH and non-physiological elution buffer used to separate PDELNs from antibodies may irreversibly affect the biological function of the collected PDELNs [68]. In addition, this method also presents challenges including low yield and high antibody cost, with chemical antibodies representing a potential direction for improvement. A current limitation is that the characteristic proteins of PDELNs are currently unclear and there is a lack of commercial antibodies [69].

### Characterization of PDELNs

**Fig. 3:**
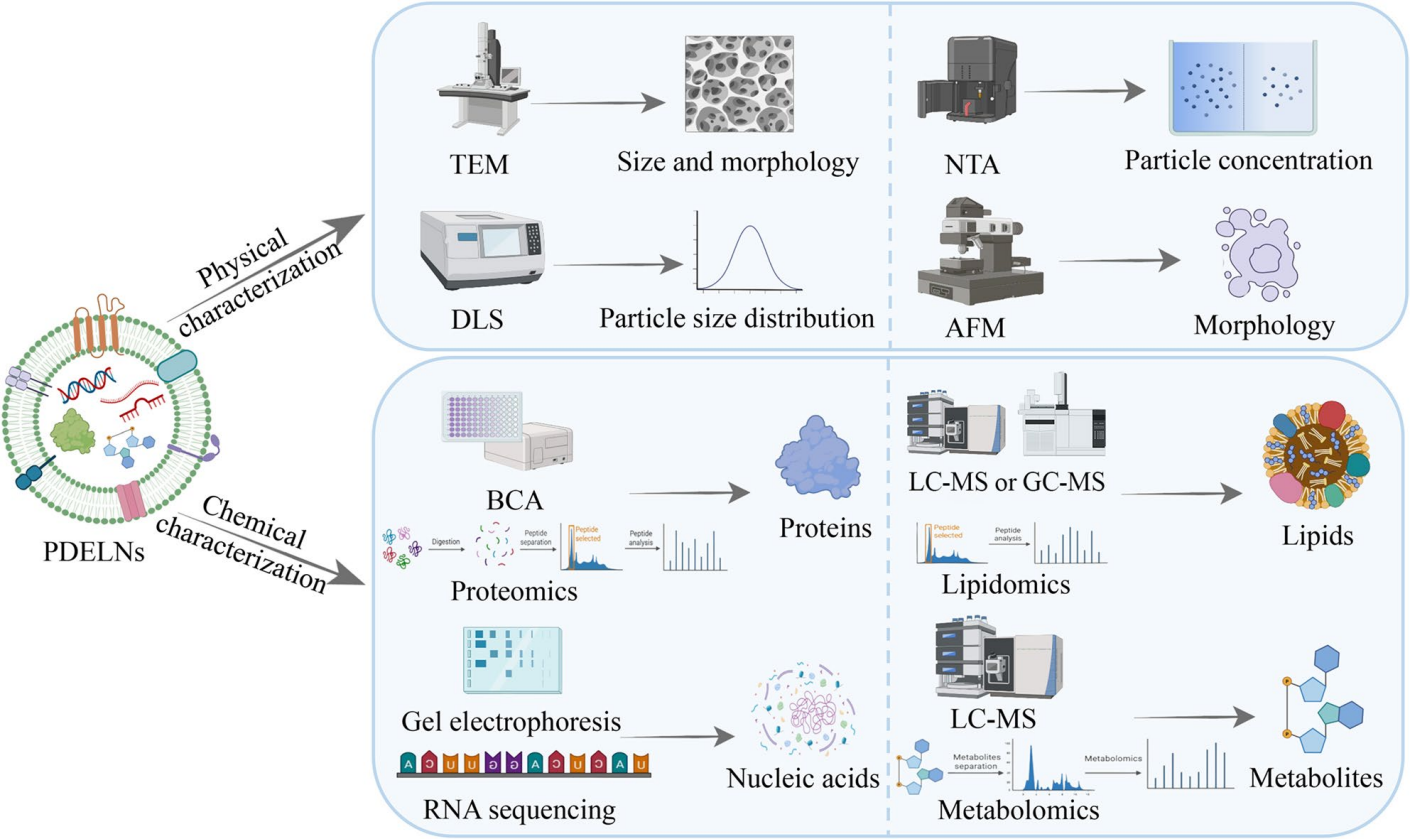
Diagram of the characterization methods for plant-derived exosome-like nanovesicles (PDELNs), mainly being divided into physical characterization and chemical characterization. Physical characterization techniques involve transmission electron microscopy (TEM), atomic force microscopy (AFM), nanoparticle tracking analysis (NTA), dynamic light scattering (DLS), etc. Chemical characterization approaches include bicinchoninic acid assay (BCA), gel electrophoresis, liquid chromatograph-mass spectrometer (LC-MS), gas chromatography-mass spectrometry (GC-MS), RNA sequencing, proteomics, lipidomics, metabolomics, etc

The research and application of PDELNs require careful consideration of preparation methods and stability. Beyond this, it is also crucial to conduct comprehensive quality control through characterization to minimize batch-to-batch variations resulting from the heterogeneity of PDELNs. The characterization of PDELNs primarily includes physical characterization for morphology observation and chemical characterization for composition inspection (Fig. 3).

### Morphology and physical characterization of PDELNs

PDELNs shares similar physicochemical properties to animal-derived exosomes, and its identification can be approached through morphology, size, particle size distribution, surface charge, and surface proteins [70]. Morphological observation typically employs electron microscopy techniques, including scanning electron microscopy (SEM), transmission electron microscopy (TEM), cryo-electron microscopy (cryo-EM), and atomic force microscopy (AFM) [71]. Its morphology often presents as circular, spherical, butterfly-shaped, cup-shaped, or saucer-shaped. The most commonly used methods for measuring the size and surface charge of PDELNs are dynamic light scattering (DLS) and nanoparticle tracking analysis (NTA) [72]. Its particle size typically ranges from 30 to 400 nm, and the Zeta potential of different PDELN samples is uniformly negative, ranging from − 1 to -49 mV. Flow cytometry is a method developed in recent years for identifying PDELNs, primarily used to detect the quantity of PDELNs [73].

### Composition and chemical characterization of PDELNs

PDELNs contains many biomolecules, including lipids, proteins, nucleic acids, and small molecule compounds [74]. Lipids are the primary constituents of PDELNs and can be categorized into phospholipids and glycerolipids. Among lipids, phospholipids predominate, including phosphatidylcholine, phosphatidic acid, phosphatidylethanolamine, phosphatidylinositol, phosphatidylglycerol, etc [75]. For comprehensive lipid characterization, liquid chromatography–mass spectrometry (LC-MS) and gas chromatography–mass spectrometry (GC-MS) are the most commonly employed analytical platforms. Further enhancing lipid analysis, ion mobility separation can be coupled with LC–MS (e.g., trapped ion mobility mass spectrometry), which effectively distinguishes isobaric and isomeric lipid species through orthogonal gas-phase separation based on ion size, shape, and charge. This approach is particularly well-suited for rapid untargeted lipidomics screening, enabling high-confidence identification of a broad range of lipid species in a single analytical run. Collectively, these techniques are regarded as standard and robust tools in lipidomics, enabling sensitive identification and accurate quantification of diverse lipid species in PDELNs [76, 78].

PDELNs contains a diverse array of proteins, primarily including those involved in cytoskeleton formation, metabolic signaling, cellular transport, and secretory pathways [79]. The protein concentration of PDELNs is typically measured using the bicinchoninic acid (BCA) assay, with further analysis of their protein composition and related characteristics conducted through proteomics [80]. There are other analytical methods, including Coomassie Brilliant Blue method, Western blotting, LC-MS, GC-MS, etc [81].

PDELNs contains multiple types of nucleic acids, including DNA, mRNA, miRNA, and non-coding RNA. DNA/RNA electrophoresis is commonly used to detect the nucleic acid bands of PDELNs, and specific functional fragments are further discovered through RNA sequencing [82]. Across different plant species, small-molecule compounds exhibit variations in both quantity and diversity, ranging from moderate to significant differences. Usually, LC-MS and metabolomics are used to analyze the metabolite types of PDELNs [83].

### Multiple mechanism of PDELNs against colitis

**Fig. 4:**
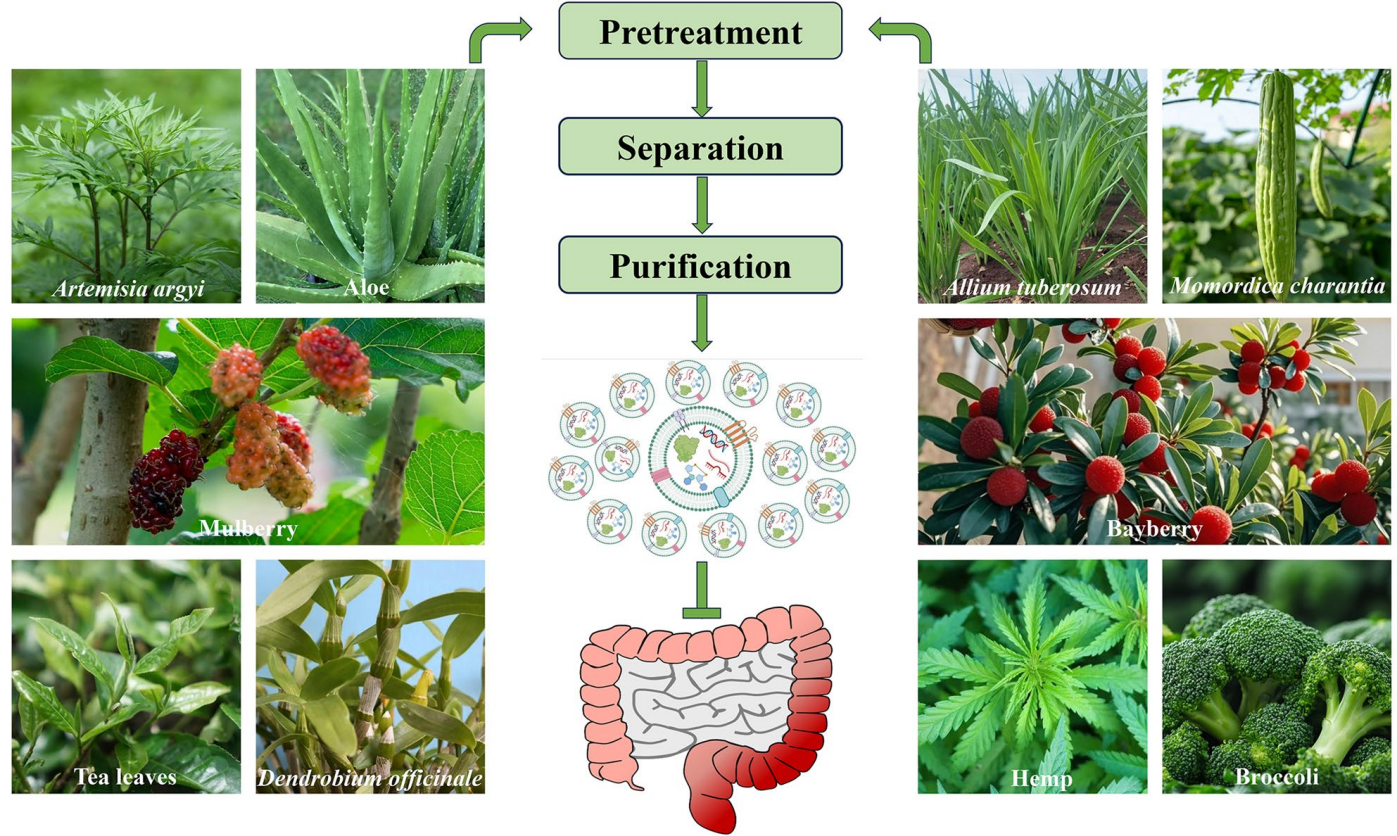
Glance at the various original plants of exosome-like nanovesicles for the treatment of ulcerative colitis in the past decade. Some of these plants have edible values, such as broccoli, tea leaves, bayberry, etc., some have medicinal values, such as *Coptis chinensis*, *Andrographis paniculata*, *Atractylodes macrocephala*, etc., and some have both edible and medicinal values, such as ginseng, turmeric, *Dendrobium officinale*, etc

**Fig. 5:**
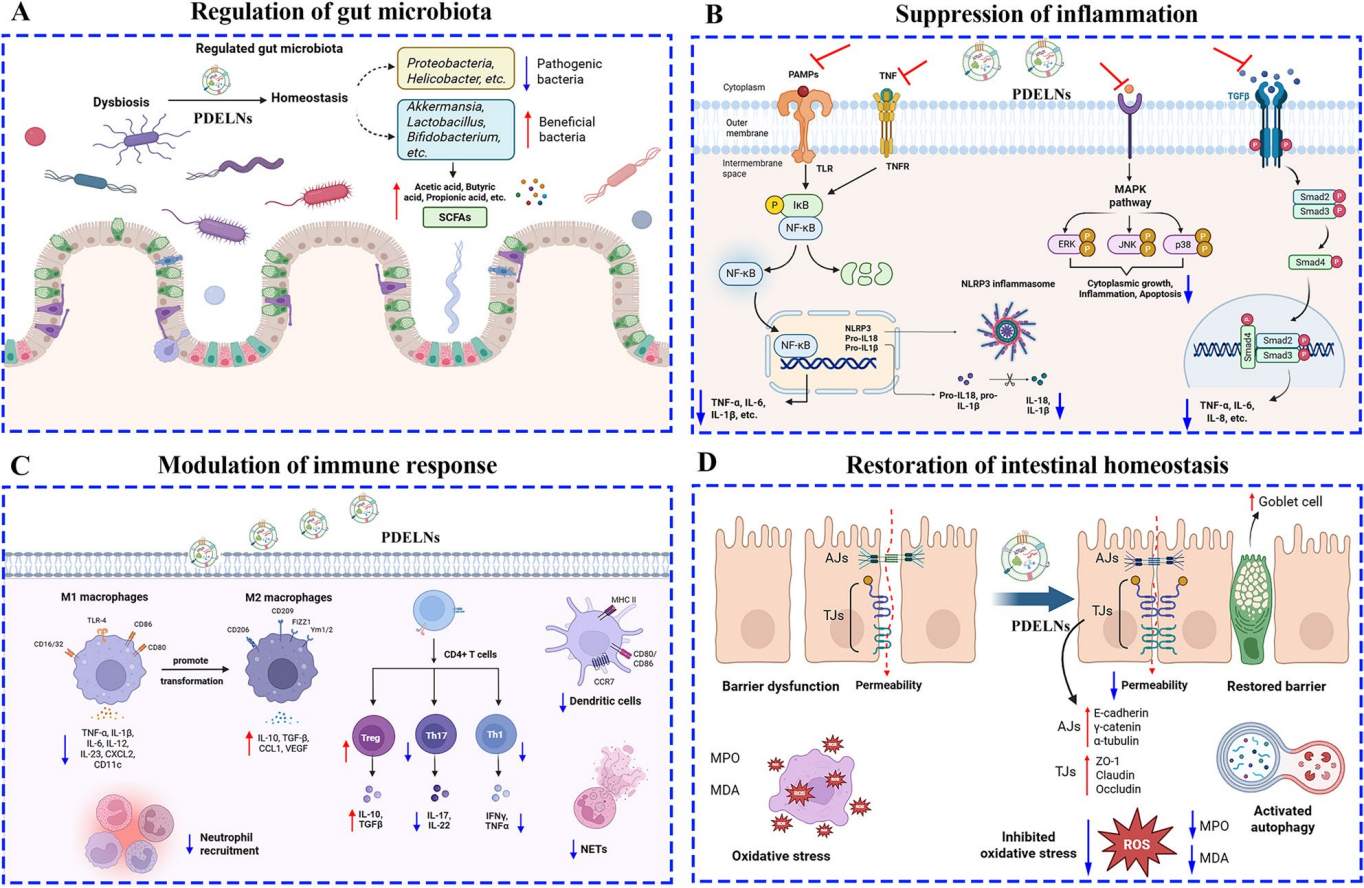
Molecular mechanisms of plant-derived exosome-like nanovesicles (PDELNs) in the treatment of colitis. (**A**) Regulation of gut microbiota. PDELNs alleviate colitis by regulating gut microbiota, characterized by a reduction in pathogenic bacteria and an increase in beneficial bacteria, which in turn elevated the levels of short-chain fatty acids (SCFAs); (**B**) Suppression of inflammation. PDELNs ameliorate colitis by suppressing inflammatory responses through the inhibition of multiple signaling pathways, including NF-κB, NLRP3, and MAPK; (**C**) Modulation of immune response. PDELNs mitigate colitis by modulating immune response through promoting the transformation of M1 macrophages to M2 macrophages, inhibiting Th17 and Th1t cells, and increasing Tregs cells; (**D**) Restoration of intestinal homeostasis. PDELNs relieve colitis by restoring intestinal barrier function, inhibiting oxidative damage, and activating autophagy. Symbols:↓, Decrease;↑, Increase; ⊥: Inhibit

Currently, a growing number of PDELNs derived from multiple plants (Fig. 4) have been shown to possess great potential in treating colitis through various molecular mechanisms. Therefore, systematic investigation of these findings is warranted. This article provides a comprehensive overview and summary of the basic information (Table 1) and regulatory mechanisms (Table 2) of PDELNs for anti-colitis. Overall, the primary mechanisms of PDELNs against colitis involve anti-inflammation, gut microbiota reshaping, and adjustment of immune response, while the secondary mechanisms of PDELNs against colitis comprise improvement of gut barrier function, anti-oxidation, and activation of autophagy (Fig. 5).

**Table 1:** Summary of the general information of plant-derived exosome-like nanovesicles (PDELNs) against colitis

Names	Plant sources	Isolation methods	Characterization methods	Particle diameter (nm)	Zeta potential (mV)	Concentration	References
GDNs	Grapefruit	DC+SDGC	EM + DLS	Range: 105.7 ~ 396.1 Average:210.8 ± 48.6	Range: -49.2~-1.5	−	[84]
GDNP	Ginger	DC+SDGC	TEM + AFM+DLS	Average: 231.6	Range: -12	−	[85]
BDNs	Broccoli	DC + SEC+SDGC	EM + DLS	Range: 18.3 ~ 118.2 Average: 32.4	Range: -39.2~-2.6 Average: -17.1	−	[86]
NTs	Tea leaves	DC+SDGC	TEM + AFM+DLS	Range: 134 ~ 145.6 Average: 140	-14.6	−	[87]
DONPs	*Dendrobium officinale*	DC+SDGC	TEM + AFM+DLS	193	-6.5	−	[88]
MBELNs	Mulberry bark	DC+SDGC	EM + NTA	Average: 151.3 ± 45.4	−	2.5 ~ 10 × 10^9^ /mL	[89]
RNVs	Hemp roots	DC	TEM + NTA	127	−	6.8 × 10^7^ /mL	[90]
SNVs	Hemp seeds	DC	TEM + NTA	180	−	1.4 × 10^8^ /mL	[90]
HSNVs	Hemp sprouts	DC	TEM + NTA	140	−	2.2 × 10^7^ /mL	[90]
LNVs	Hemp leaves	DC	TEM + NTA	107	−	6.6 × 10^7^ /mL	[90]
TDNPs	Turmeric	UC+SDGC	TEM + AFM+ DLS	177.9	-21.7	−	[91]
TNVs	Turmeric	DC+SDGC	TEM + DLS	Average: 243.9 ± 13.9	-15	−	[92]
GEN	*Panax ginseng*	DC + UF+SDGC	TEM + DLS+NTA	Average: 146.5	-19.2	1.6 ± 0.3 mg/mL	[93]
GDNPs	*Panax ginseng*	DC+SDGC	TEM + NTA	Average: 256	-39	−	[94, 95]
GDELNs	Garlic	DC+SDGC	TEM + NTA	204.7 ± 42.6	−	−	[96]
GENs	Garlic	SDGC + UC	TEM	Range: 100 ~ 200 Average: 160.2 ± 3.5	-10.1 ± 0.8	2.3 × 10^8^ /mg	[97]
GELNs	Garlic	UC+SDGC	TEM+NanoFCM	Range: 50 ~ 150 Average: 79.6	−	1.2 × 10^11^ /mL	[98]
PELNs	*Portulaca oleracea* L	DC+SDGC	TEM + DLS+NTA	Range: 30 ~ 400 Average: 180	-31.4	−	[99]
NPs	Black tea	UF + SEC	TEM + DLS+NTA	187 ± 7	-32.4 ± 2.5	4 × 10^10^ /mg	[100]
BELNs	Bayberry	DC + UF+SDGC	TEM + NTA	Range: 100 ~ 300 Average: 232.1 ± 3	−	3.2 ± 0.2 mg/mL	[101]
VNVs	*Aloe vera*	DC	TEM + NTA	151 ± 0.1	−	3.8 ± 0.2 µg/µL	[102]
ANVs	*Aloe arborescens*	DC	TEM + NTA	210 ± 0.2	−	0.4 ± 0.0 µg/µL	[102]
SNVs	*Aloe saponaria*	DC	TEM + NTA	197 ± 0.1	−	2.4 ± 0.1 µg/µL	[102]
ADNs	*Allium tuberosum*	UF + PBP	TEM + SEM+NTA	Range: 101 ~ 140 Average: 125.2 ± 4.1	−	3.8 ± 1.6 × 10^9^ /g	[103]
HELNs	*Houttuynia cordata*	DC+SDGC	TEM + NTA+DLS	Range: 58 ~ 228 Average: 100	-10.1	−	[104]
iLNVs	Lemon	UC + UF	SEM + NTA	Average: 172.8 ± 0.3	−	−	[105]
PLDENs	*Pueraria lobata*	DC + UF	NTA + DLS	Average: 75.5 ± 10.2	-21.8	−	[106]
BJ-NPs	*Boehmeria japonica*	DC + UF+SDGC	SEM + DLS	Average: 180 ± 22.4	−	1 mg/mL	[107]
MCEVs	*Momordica charantia*	DC+SDGC	TEM + DLS+NTA	Average: 164.3 ± 68.2	-28.1 ± 3.8	−	[108]
FAELNs	*Folium Artemisiae Argyi*	UC + SEC	TEM + DLS	Range: 68 ~ 290 Average: 203	-10.5	−	[109]
AMEVLP	*Atractylodes macrocephala*	DC + UF	TEM+NanoFCM	Average: 90.6 ± 19.7	−	6.3 × 10^11^ /mL	[110]
CELNs	*Zanthoxylum bungeanum*	DC	TEM + NTA	Range: 100 ~ 300 Average: 164	−	1.1 × 10^11^ /mL	[111]
RELNs	*Zanthoxylum bungeanum*	DC	TEM + NTA	Range: 100 ~ 300 Average: 162	−	7 × 10^10^ /mL	[111]
Cc-ELNs	*Coptis chinensis*	DC	TEM+NanoFCM	Average: 55 ~ 120	−	8.5 × 10^10^ /mL	[112]
PM-EVLPs	*Prunus mume*	DC+SDGC	TEM + NTA	Range: 124	-27	−	[113]
APELNs	*Andrographis paniculata*	DC+SDGC	TEM + DLS	Range: 50 ~ 400 Average: 180	-40	−	[114]
TPELNs	Tangerine	DC + PBP	TEM + DLS+NTA	Average: 209.5 ± 8.3	-15.9 ± 0.2	2.7 × 10^10^ /mL	[115]

**Table 2:** Molecular mechanisms of plant-derived exosome-like nanovesicles (PDELNs) against colitis

Names	Plant sources	Models	Dosages	Active components	Mechanisms	References
GDNs	Grapefruit	DSS-evoked colitis C57BL/6J mice	10 mg/kg	Phosphatidylcholine, phosphatidic acid	Up: HO-1, IL-10 Down: TNF-α, IL-1β, IL-6, MCP-1, CXCL9, CXCL10, KC	[84]
GDNP	Ginger	DSS-evoked colitis FVB/NJ mice	0.3 mg per mouse	6-gingerol, 6-shogaol	Up: IL-10, IL-22 Down: TNF-α, IL-1β, IL-6	[85, 116]
BDNs	Broccoli	DSS-evoked colitis C57BL/6 mice	250 µg per mouse	/	Up: IL-10, IL-4, TGF-β, Aldh1a2, p-AMPK, PD-L1, PD-L2, B7H3, IL-10 + CD4+ T cells, PD-1 Down: TNF-α, IL-17 A, IFN-γ, IL-12, IL-23, CCL2, CCL20, CXCL1, CCR2, CCR6, CCR9, CD80, CD86, CD40, CD11b+CD11c+MHCII + DCs, CD69, CD25, p-S6, p70S6K	[86]
NTs	Tea leaves	LPS-treated Raw264.7 cells DSS-evoked colitis C57BL/6 mice	8 µg/mL 2 mg/kg	Phosphatidic acid, phosphatidylcholine, gallic acid, caffeine, epigallocatechin gallate, epicatechin gallate, epicatechin, myricetin, quercetin, hyperoside	Up: IL-10, ZO-1, Mucin-2, GSH, HO-1, Lachnospiraceae, *Bifidobacterium*, *Akkermansia* Down: TNF-α, IL-6, IL-12, ROS, MPO, MDA, Firmicutes*/*Bacteroidetes, Proteobacteria, *Oscillibacter*, *Helicobacter*, *Mucispirillum*, *Brachyspira*, *Desulfovibriom*, *Parasutterella* spp.	[87]
DONPs	*Dendrobium officinale*	LPS-treated Raw264.7 cells DSS-evoked colitis FVB mice	2.5, 5, 10, 20 µg/ml 0.5, 1 mg/kg	/	Down: TNF-α, ROS, MPO, WBC, lymph number, Gran number, Mon number, Chao index, Ace index, Firmicutes/Bacteroidetes, Proteobacteria	[88]
MBELNs	Mulberry bark	MC38 or Caco-2 cells DSS-evoked colitis C57BL/6J mice	5 × 10^9^ particles/mL 10 × 10^9^ particles/mouse	/	Up: AhR, COPS8, pAhR, luciferase, Cytochrome P450, family 1, subfamily A, CYP1A1, IDO1, ZO-1, FITC-dextran, Defa-b1, Ang-4, Defa-rs1, RegIIIγ, Dehalobacteriaceae, Lachnospiraceae, Ruminococcaceae, Erysipelotrichaceae, Mogibacteriaceae, Porphyromonadaceae, Prevotellaceae, Rikenellaceae, Paraprevotellaceae, Pseudomonadaceae, Desulfovibrionaceae, Verrucomicrobiaceae Down: IL-1β, IL-6, N-CUL1/CUL1, NF-κB, IKK-β, Bacteroidaceae, S24.7, Odoribacteraceae, Lactobacillaceae, Turicibacteraceae, Clostridiaceae, Alcaligenaceae, Helicobacteraceae, Mycoplasmataceae, *Corynebacterium*, *Listeria (L.) monocytogenes-EGD*	[89]
RNVs	Hemp roots	DSS-treated T84 cells DSS-evoked colitis C57BL/6 mice	1, 10 µg/mL 1 mg/kg/d	/	Up: Claudin-4, Occludin, ZO-1, E-cadherin, α-tubulin, TEER, Cyclin D1, eNOS Down: TNF-α, NO, p-NF-κB/NF-κB, p-IκB/IκB, COX-2, 3-NT, iNOS, ALT, AST, FITC-D4	[90]
SNVs	Hemp seeds	DSS-treated T84 cells DSS-evoked colitis C57BL/6 mice	1, 10 µg/mL 1 mg/kg/d	/	Up: Claudin-4, ZO-1, α-tubulin, Cyclin D1, eNOS Down: TNF-α, NO, p-NF-κB/NF-κB, ALT, AST	[90]
HSNVs	Hemp sprouts	DSS-treated T84 cells DSS-evoked colitis C57BL/6 mice	1, 10 µg/mL 1 mg/kg/d	/	Up: Claudin-4, ZO-1, α-tubulin, Cyclin D1, eNOS Down: TNF-α, p-NF-κB/NF-κB, COX-2, ALT, AST	[90]
LNVs	Hemp leaves	DSS-treated T84 cells DSS-evoked colitis C57BL/6 mice	1, 10 µg/mL 1 mg/kg/d	/	Up: Occludin, E-cadherin, Cyclin D1, eNOS Down: TNF-α, p-NF-κB/NF-κB, COX-2, ALT, AST	[90]
TDNPs	Turmeric	LPS-treated Colon-26/ RAW264.7 cells DSS-evoked colitis FVB/NJ mice	20 ~ 200 µg/mL 3 mg/dose	Curcumin	Up: HO-1, goblet cells, E-cadherin Down: TNF-α, IL-1β, IL-6, MPO, Lipocalin-2, p-NF-κB-p65, NF-κB-p65 (nucleus)	[91]
TNVs	Turmeric	NCM 460/HT-29 cells LPS-treated Raw264.7 cells DSS-evoked colitis ICR mice	25 µg/mL 10 mg/kg	Curcumin	Up: CD206 + macrophages, IL-10, E-cadherin, ZO-1, Occludin, *Akkermansia*, *Lactobacillus*, *Clostridia_UCG-014*, *Bifidobacterium* Down: TNF-α, IL-1β, IL-6, IL-12p70, IL-1α, IFN-β, MPO, MCP-1, F4/80 + CD11b+ macrophage, CD16/32 + macrophage, *Bacteroides*, *Escherichia-Shigella*, *Helicobacter*, *Staphylococcus*	[92]
GEN	*Panax ginseng*	LPS-treated Caco-2/RAW264.7 cells DSS-evoked colitis Balb/C mice	1 ~ 50 µg/mL 1 mg per mouse	Ginsenosides	Up: CD 206 + macrophage, PDL1, IL-10, *Lactobacillus*, *Alistipes* Down: TNF-α, IL-17A, IL-6, NF-κB, IL-1β, IL-8, iNOS, Firmicutes/Bacteroidota, *Helicobacter*, *Oscillibacter*, *Ruminococcus*	[93]
GDNPs	*Panax ginseng*	LPS-treated RAW264.7/Caco-2 cells DSS-evoked colitis C57BL/6J mice	5, 10, 20 µg/mL 5, 10 mg/mL (0.2 mL per mouse)	Ginsenosides	Up: JC-1, MMP, ZO-1, Claudin-1, Occludin, IL-10, SOD, Keap1, Nrf2, HO-1, Nrf2 (nuclear), Lgr5, Wnt 3 A, TGF-β1, β-catenin, Ki67, GCLM, GCLC, NQO1, BMI-1, CDX1, Mucin-2, p-p62/p62, Chao index, Shannon index, Observed species, Bacilli Down: DHE, TNF-α, IL-1β, IL-6, PGD_2_, PGE_2_, TLR4, MYD88, NO, p-ERK, p-JNK, p-p38, ROS, MDA, Firmicutes/Bacteroidetes	[94]
GDNPs	*Panax ginseng*	LPS-treated RAW264.7/Caco-2 cells DSS-evoked colitis C57BL/6J mice	5, 10, 20 µg/mL 5, 10 mg/mL (0.2 mL per mouse)	Ginsenosides	Up: CD206, TGF-β, GFP-LC3 spots, autophagic lysosomes, Beclin1, Atg7, LC3II/ LC3I, IL-10, ZO-1, Occludin, LC3, DAO (tissue), 5-HT (tissue), SP (tissue) Down: CD86, iNOS, p-mTOR, p-AKT, TNF-α, IL-1β, NO, NF-κB, NF-κB (nuclear), P-IKKα/β, P-IĸBα, COX-2, FITC, DAO (serum), 5-HT (serum), SP (serum), D-LA (plasma)	[95]
GDELNs	Garlic	DSS-evoked colitis C57BL/6 mice	2.5 mg/mouse	/	Up: TGF-β1 Down: TNF-α, IL-6, IL-8, p-Smad2, p-Smad3	[96]
GENs	Garlic	LPS-treated Caco-2 cells DSS-evoked colitis C57BL/6J mice	1, 5, 10 µg/mL 20, 100, 500 mg/kg	Han-miR3630-5p, phosphatidylcholine	Up: ZO-1, Occludin, Claudin-1, acetic acid, butyric acid, propionic acid, valeric acid, isobutyric acid, isovaleric acid, total acids, Firmicutes, Lachnospiraceae Down: TNF-α, IFN-γ, IL-1β, IL-6, IL-17 A, NO, TLR4, MyD88, p-p65, Proteobacteria, *Helicobacter*, *Escherichia-Shigella*, *Akkermansia*	[97]
GELNs	Garlic	DSS-evoked colitis C57BL/6J mice	2, 10, 50 mg/kg	peu-MIR2916-p3	Up: ZO-1, Occludin, Mucin-2, *Eubacterium ruminantium*, *Insolitispirillum*, *Megasphaera*, *Acetatifactor*, *Bacteroides*, *Bacteroides thetaiotaomicron* Down: TNF-α, IL-6, *Anaerostipes*, *Anaeroplasma*, *Ileibacterium*, *Dubosiella*	[98]
PELNs	*Portulaca oleracea* L	DSS-evoked colitis C57BL/6 mice	50, 100 mg/kg	/	Up: IL-10, CD4^+^CD8^+^T cells, Firmicutes, *Patescibacteria*, Lachnospiraceae, Ruminococcaceae, Muribaculaceae, Lactobacillaceae, Saccharimonadaceae, Erysipelotrichaceae, Prevotellaceae, Clostridiales_vadinBB60_group, Burkholderiaceae, Vibrionaceae, Eggerthellaceae, Peptococcaceae, Bacillaceae, Lachnospiraceae_NK4A136_group, *Lactobacillus*, Ruminococcaceae_UCG-014, *Candidatus_Saccharimonas*, Ruminiclostridium_6, *Dubosiella*,* Parasutterella*,* Alloprevotella*, GCA-900,066,575, *Ileibacterium*,* Photobacterium*,* Turicibacter*, Lachnospiraceae_UCG-006, *Ruminococcus_1*,* Bilophila*,* Enterorhabdus*,* Butyricicoccus*, ASF356, UBA1819, *Harryflintia*, GCA-900,066,225, *Muribaculum*,* Acetatifactor*,* Lactobacillus_gasseri*,* Lactobacillus_reuteri*,* Alistipes_inops*,* Ileibacterium_valens*,* Photobacterium_damselae_*subsp*_damselae*,* Ruminococcus_flavefaciens*, indole derivatives Down: TNF-α, IL-1β, IL-6, IL-12, MPO, Zbtb7b, Proteobacteria, Deferribacteres, Enterobacteriaceae, Deferribacteraceae, Enterococcaceae, Tannerellaceae, *Escherichia-Shigella*,* Mucispirillum*,* Enterococcus*, Rikenellaceae_RC9_gut_group, *Parabacteroides*,* Bacteroides_thetaiotaomicron*,* Mucispirillum*_sp_69, *Enterococcus_faecalis*,* Parabacteroides_goldsteinii*	[99]
NPs	Black tea	DSS-evoked colitis C57BL/6 mice	5 mg/kg	Caffeine, gallic acid, epigallocatechin gallate, epicatechin gallate	Up: E-cadherin, ZO-1, Claudin-1 Down: TNF-α, IL-1β, IL-6, p-STAT3	[100]
BELNs	Bayberry	LPS-treated Raw264.7 cells DSS-evoked colitis C57BL/6J mice	8 µg/mL 100 µL	/	Up: ZO-1, Mucin-2, IL-10 Down: TNF-α, MPO	[101]
VNVs	*Aloe vera*	T84 cells DSS-evoked colitis C57BL/6 mice	1, 10 µg/mL 1 mg per mouse	/	Up: ZO-1, Claudin-4, Occludin, γ-catenin, α-tubulin, E-cadherin, FITC-dextran Down: LPS, TNF-α, NO, p-NF-κB, p-IκB, COX-2, 3NT, TEER	[102]
ANVs	*Aloe arborescens*	T84 cells DSS-evoked colitis C57BL/6 mice	1, 10 µg/mL 1 mg per mouse	/	Up: ZO-1, Claudin-4, Occludin, γ-catenin, α-tubulin, E-cadherin, FITC-dextran Down: LPS, TNF-α, NO, p-NF-κB, p-IκB, COX-2, 3NT, TEER	[102]
SNVs	*Aloe saponaria*	T84 cells DSS-evoked colitis C57BL/6 mice	1, 10 µg/mL 1 mg per mouse	/	Up: ZO-1, Claudin-4, Occludin, γ-catenin, α-tubulin, E-cadherin, FITC-dextran Down: LPS, TNF-α, NO, p-NF-κB, p-IκB, COX-2, 3NT, TEER	[102]
ADNs	*Allium tuberosum*	LPS-treated Raw264.7 cells DSS-evoked colitis C57BL/6 mice	0.5, 1, 5, 10 ng/mL 1.25 mg/kg	/	Up: IL-10, ZO-1, Occludin, acetic acids, butyric acids, Bacteroidaceae, Lactobacillaceae, *Lactobacillus*, *Limosilactobacillus* Down: NO, TNF-α, IL-1β, IL-6, amyloid A, p-NF-κB, p-IκB, Shannon index, Firmicutes/Bacteroidota, Proteobacteria, Clostridia_UCG, Ruminococcaceae, Lachnospiraceae_UCG006	[103]
HELNs	*Houttuynia cordata*	DSS-evoked colitis C57BL/6 mice or NLRP3^−/−^mice	5, 10 mg per mouse	Isoquercitrin, procyanidin B2, luteolin, glutamine, trigonelline, diosgenin	Up: IL-10, ZO-1, Mucin-2, Occludin, Claudin-1, goblet cells, CD206+, Chao 1, Shannon index, Firmicutes, Actinobacteriota, Lactobacillaceae, Eggerthellaceae, Oscillospiraceae, *Lactobacillus*, *Odoribacter*, *Enterorhabdus*, *Solibacillus*, *Lactobacillus murinus*, *Lactobacillus apodeme* Down: F4/80 + CD11b+, CD80+, CD11c+, CD11b + Gr-1+, TNF-α, IL-1β, IL-6, IL-17, IL-18, IL-23, Pro-IL-1β, Cle-IL-1β, Caspase-1, NLRP3, Bacteroidota, Bacteroidaceae, *Escherichia-Shigella*, *Helicobacter*, *Candidatus_Saccharimonas*, *Staphylococcus*	[104]
iLNVs	Lemon	LPS-treated THP-1 M0 cells DNBS-evoked colitis Wistar rats	2.5, 5 µg/mL 0.6 mg/kg	Eriocitrin, hesperidin	Up: IL-10, Nrf2, Occludin, Lachnospiraceae NK4B4 Down: IL-6, TNF-α, MPO, NF-κB, *Enteractinococcus*, *Acetatifactor*	[105]
PLDENs	*Pueraria lobata*	DSS-evoked colitis C57BL/6 mice	0.5, 2, 5 mg/kg/d	/	Up: IL-10, YM1, CD206, ZO-1, Mucin-2, Occludin, sobs, chao, ace, shannon, norank_f_Muribaculaceae, Muribaculaceae, Lactobacillaceae, Lachnospiraceae, Akkermansiaceae, Eggerthellaceae, *Lactobacillus*, *Akkermansia*, norank_o_Clostridia_UcG-014, Lachnospiraceae_NK4A136_group, norank_f_norank_o_Clostridia_UCG-014 Down: IL-6, IL-1β, TNF-α, CCL2, CD11c, TGF-β1, simpson, Proteobacteria, Verrucomicrobiota, Campilobacterota, Cyanobacteria, Rikenellaceae, Enterobacteriaceae, Bacteroidaceae, Oscillospiraceae, Clostridiaceae, Sutterellaceae, Tannerellaceae, Helicobacteraceae, norank_o_Rhodospirillales, *Escherichia-Shigella*, *Alistipes*, *Bacteroides*, Rikenellaceae_Rc9_gut_group, *Dubosiella*, norank_f_Oscillospiraceae, Prevotellaceae_UCG-001, *Faecalibaculum*, Eubacterium_fissicatena_group	[106]
BJ-NPs	*Boehmeria japonica*	LPS-treated BMDCs DSS-evoked colitis C57BL/6 mice	10, 50, 100, 200 µg/mL 1 mg per mouse	/	Up: IL-10, Treg, Apoptosis, CD4 + T cells, CD3 + CD4+Foxp3+ Down: TNF-α, IL-12p70, IFN-γ, IL-17 A, CD80, CD86, MHC-II, CD3 + CD4+IFN-γ+, CD3 + CD4+IL-17 A+	[107]
MCEVs	*Momordica charantia*	LPS/H_2_O_2_-treated RAW264.7 cells DSS-evoked colitis C57BL/6 mice	5, 10 µg/mL 15, 30 mg/kg	Phosphatidylcholine, β-carotene, lycopene, astaxanthin	Up: IL-10, Bcl-2, ZO-1, Occludin, Claudin-1, goblet cells, Chao index, shannon index, *Muribaculum*, *Alistipes*, Indole-3-methanol, Indole, Indoleacetic acid Down: ABTS radical, DPPH radical, ·OH, O₂ˉ·, Apoptosis, Bax, TNF-α, IL-1β, IL-6, ROS, MPO, CD86, leucocytes, neutrophil, Verrucomicrobia, Firmicutes/Bacteroidota, *Escherichia-Shigella*, Indoxyl sulfate	[108]
FAELNs	*Folium Artemisiae Argyi*	LPS-treated HT-29/ NCM460/RAW264.7 cells DSS-evoked colitis Balb/C mice	1, 10, 20, 50, 100 µg/mL; 25, 50 mg/kg	Phosphatidylcholine, raffinose, artemisinin, canthin-6-one	Up: Bcl-2, ZO-1, E-cadherin, Occludin, FITC-dextran, goblet cells, CD206 + macrophage, sob index, Lachnospiraceae, *Roseburia*, *Oscillibacter* Down: Bax, IL-6, IL-1β, TNF-α, MCP-1, IFN-γ, ROS, MPO, Firmicutes/Bacteroidota, Proteobacteria, Prevotellaceae, *Erysipelatoclostridium*	[109]
AMEVLP	*Atractylodes macrocephala*	LPS-treated RAW264.7 Cells DSS-evoked colitis C57BL/6J mice	0.1, 0.25, 0.5 µg/mL; 0.5, 2 mg/kg	Atractylenolide, ginsenoside F2, resveratrol, artemisinin	Up: IL-10, Occludin, ZO-1, Claudin, goblet cells, OTUs, Chao1 index, shannon index, Firmicutes, *Bacteroides*, Muribaculaceae, Rikenellaceae_RC9_gut_group, Lachnospiraceae_NK4A136_group, *Alistipes*, Thick-walled Bacteria, Bacteridgk, Bacteroidetes, 6-Hydroxymelatonin, L-Kynurenine, Melatonin, Indole-3-acetamide, hydrocortisone, L-Tyrosine, Octopine, Thymidine, Cinchophen, Valylproline, Leucylproline, 1-Methyladenosine Down: IL-1β, IL-6, IL-12, TNF-α, Proteobacteria, *Escherichia-Shigella*	[110]
CELNs	*Zanthoxylum bungeanum*	LPS-treated THP-1 cells DSS-evoked colitis C57BL/6 mice	1 × 10^8^ particles/mL 1 × 10^11^ particles/kg	miRNA-1, miRNA-21	Up: ZO-1, Occludin, Claudin-1, 3-(2-hydroxyphenyl) propanoic acid, miRNA-1, miRNA-21 Down: IL-1β, IL-6, IL-8, TNF-α	[111]
RELNs	*Zanthoxylum bungeanum*	LPS-treated THP-1 cells DSS-evoked colitis C57BL/6 mice	1 × 10^8^ particles/mL 1 × 10^11^ particles/kg/d	miRNA-1, miRNA-21, phosphatidylcholine	Up: ZO-1, Occludin, Claudin-1, miRNA-1, miRNA-21, Mesaconate, 6-hydroxyhexanoic acid Down: IL-1β, IL-6, IL-8, TNF-α	[111]
Cc-ELNs	*Coptis chinensis*	DSS-evoked colitis C57BL/6J mice	50 µg per mouse 5 nmol per mouse	miR-5106	Up: IECs proliferation, ISCs proliferation, Lgr5^+^ ISCs, goblet cell, Ki67 Down: *Slc39a2*, GSDMD, caspase-1, MPO, ROS, NETs, neutrophil	[112]
PM-EVLPs	*Prunus mume*	LPS-treated BMDMs PMA-stimulated THP-1 cells DSS-evoked colitis C57BL/6 mice TNBS-induced colitis BALB/c mice	0.5, 1.5 × 10^10^ particles/mL	miR159	Down: NEK7-NLRP3 interaction, ASC oligomerization, NEK7, CD11b+ Ly6G+ neutrophils, MPO, cleaved caspase-1, cleaved IL-1β, IL-1β, IL-18	[113]
APELNs	*Andrographis paniculata*	LPS-treated NCM460, HCT116, and RAW264.7 cells DSS-evoked colitis C57BL/6 or IL-10^−/−^ mice	20, 800 µg/L 200 µL, 2.5 mg/mL	/	Up: IL-4R, Claudin-1, ZO-1, Mucin-2, Occludin, CD206+, Firmicutes, Lactobacillaceae, Staphylococcaceae, *Staphylococcus*, *Lactobacillus*, *Lactobacillus murinus* Down: MPO, IL-12, IL-1β, TNF-α, IL-6, FITC-dextran, CD86+, *Aeromonas veronii* B565	[114]
TPELNs	Tangerine peel	LPS-treated RAW264.7 cells DSS-evoked colitis C57BL/6J mice	10^5^, 10^6^, 10^7^, 10^8^, 10^9^ particles/mL 10^8^, 10^9^, 10^10^ particles/mL	/	Up: IL-10, Firmicutes/Bacteroidota, Firmicutes, *Lactobacillus*, *Bifidobacteria*, *Bifidobacterium*, norank_o_Gastranaerophilales, UCG-009, ginsenoside Ro, indole carboxylic acid sulfate, sulfocholic acid, indole-3-carboxylic acid-O-sulfate Down: IL-1β, TNF-α, iNOS, Bacteroidota, *Escherichia−Shigella*	[115]

### Inhibition of inflammation

Ulcerative colitis is fundamentally characterized by chronic, relapsing inflammation of the colonic mucosa, predominantly affecting the rectum and extending proximally [117]. This inflammation arises from a dysregulated immune response to commensal gut microbiota in genetically predisposed individuals. Key mediators include elevated levels of pro-inflammatory cytokines (e.g., TNF-α, IL-6, IL-1β, and IL-23), which drive neutrophil infiltration, epithelial injury, and crypt abscess formation [118]. The inflammatory cascade is perpetuated by sustained activation of the NF-κB and STAT3 signaling pathways, leading to mucosal ulceration, loss of goblet cells, and impaired wound healing [119]. Histologically, ulcerative colitis exhibits diffuse, continuous inflammation limited to the mucosal and submucosal layers, distinguishing it from Crohn’s disease [120]. Therapeutic strategies targeting inflammation (e.g., anti-TNF biologics, JAK inhibitors) underscore its central role in ulcerative colitis pathogenesis [121].

In the study from Sriwastva et al., they isolated mulberry bark-derived exosome-like nanoparticles (MBELNs) using a combination of DC and SDGC, and explored the possible mechanism of MBELNs against colitis [89]. The results showed that MBELNs could induce the production of a series of antimicrobial peptides (AMPs), activate the AhR/COP9/COPS8 signaling pathway, resist the invasion of various inflammatory signals, and achieve the goal of treating colitis.

Liu et al. isolated a specific subset of turmeric-derived nanoparticles, termed TDNPs, by the conjunction of UC and SDGC [91]. The experimental proofs indicated that TDNPs ameliorated mice colitis by restraining the levels of pro-inflammatory factors covering TNF-α, IL-6, and IL-1β and antioxidative effect. Mechanistic studies further indicated that the protective effects of TDNPs were partially mediated through the inactivation of the NF-κB signaling pathway. In addition, some other PDELNs (e.g., ANVs, SNVs and VNVs from Aloe, ADNs from *Allium tuberosum*, iLNVs from lemon) have also been reported to exert anti-inflammatory effects in colitis models, largely via suppression of the NF-κB pathway [102, 103, 105].

Similarly, Gao et al. prepared turmeric-derived nanovesicles (TNVs) via DC and SDGC [92]. Their study revealed that TNVs exerted protective effects against colitis primarily through anti-inflammatory mechanisms. TNVs significantly elevated the level of the anti-inflammatory cytokine IL-10 while reducing the expression of multiple pro-inflammatory mediators, including IL-1β, MCP-1, TNF-α, IL-6, IL-12p70, IL-1α, and IFN-β.

Among the study of Kim et al., they obtained ginseng-derived exosome-like nanoparticles (GEN) by the triple integration of DC, UF and SDGC, and plumbed the action and mechanism of GEN against colitis [93]. The results displayed that GEN alleviated colitis by modulating the inflammatory response, including the downregulation of pro-inflammatory cytokines such as TNF-α, IL-17 A, and IL-6, and the upregulation of the anti-inflammatory cytokine IL-10. Further mechanism experiment certificated that the modulation of GEN towards the inflammatory reaction is closely related to the inhibition of NF-κB. Based on the experiment of Yang et al., they prepared ginseng-derived nanoparticles (GDNPs) by the application of DC and SDGC, and explored the action mechanism of GDNPs treating colitis [94]. The results indicated that the anti-colitis role of GDNPs is quietly associated with the adjustment of inflammation. GDNPs could restrain the inflammatory reaction via lowering the levels of pro-inflammatory factors TNF-α, IL-1β, IL-6, PGD_2_, PGE_2_, rising the amount of anti-inflammatory molecule IL-10, and suppressing the TLR4/MAPK pathway. In a follow-up study, Yang et al. further revealed that GDNPs up-regulated IL-10 while downregulating IL-1β, COX-2c and iNOS, and targeted the IKK/IкB/NF-кB signaling axis to relieve intestinal inflammation [95].

In the study of Xiao et al., they acquired garlic-derived exosome-like nanoparticles (GDELNs) by the conjunction of DC and SDGC, and inspected the role and mechanism of GDELNs on colitis [96]. GDELNs upregulates the expression of TGF-β1 by regulating the metabolic products of gut microbiota, thereby activating the downstream Smad2/Smad3 signaling pathway to regulate inflammatory cytokines (e.g., TNF-α, IL-6 and IL-8), ultimately alleviating colitis.

During the experiment of Zhu et al., they gained the garlic-derived exosome-like nanovesicles (GENs) by the combination of SDGC and UC, and inquired into the effect and mechanism of GENs towards colitis [97]. The results demonstrated that GENs played an anti-inflammatory role via suppressing nitric oxide overproduction and reducing the levels of pro-inflammatory cytokines, including TNF-α, IL-1β, IL-6 and IFN-γ. Mechanistic investigations suggested that their protective effects were associated with inhibition of the TLR4/MyD88/NF-κB pathway and maintenance of intestinal microbiota balance.

In accordance with the research of Han et al., they obtained black tea nanoparticles (NPs) by the integration of UF and SEC, and inspected the action and mechanism of NPs on colitis [100]. The proofs exhibited that NPs might alleviate DSS-elicited colitis through inhibiting the amounts of pro-inflammatory factors TNF-α, IL-1β, IL-6 and the expression of inflammation-related protein p-STAT3.

Among the experiment of Li et al., they isolated *Houttuynia cordata*-derived exosome-like nanoparticles (HELNs) by the combination of DC and SDGC, and inspected the effect and mechanism of HELNs against colitis [104]. The results showed that the anti-colitic role of HELNs is tightly related to anti-inflammation. HELNs evidently improved the expression of anti-inflammatory factor IL-10, and lowered the levels of pro-inflammatory factors TNF-α, IL-1β, IL-6, IL-17, IL-18, IL-23, Pro-IL-1β and Cle-IL-1β. Further studies suggested that these effects were mediated via the inhibition of NLRP3/NOD-like receptor signal pathway.

Lv et al. prepared *Prunus mume* derived extracellular vesicle-like particles (PM-EVLPs) by the integrated approach of DC and SDGC [113]. Oral administration of PM-EVLPs significantly ameliorated both DSS- and TNBS-induced colitis in mice. Mechanistically, PM-EVLPs selectively disrupted the NEK7-NLRP3 interaction, thereby inhibiting NLRP3 inflammasome assembly and activation in macrophages. This led to reduced cleavage of caspase-1 and subsequent suppression of the pro-inflammatory cytokines IL-1β and IL-18, ultimately mitigating intestinal inflammation.

In addition to the above, other PDELNs, including NTs from tea leaves, PELNs from *Portulaca oleracea* [99], ADNs from *Allium tuberosum* [103], iLNVs from lemon [105], BJ-NPs from *Boehmeria japonica* [107], MCEVs from *Momordica charantia* [108], FAELNs from *Folium Artemisiae Argyi* [109], AMEVLP from *Atractylodes macrocephala* [110], ZbELNs from *Zanthoxylum bungeanum* [111], TPELNs from tangerine [115], have also demonstrated anti-inflammatory effects in colitis models. These effects were primarily mediated through upregulation of the anti-inflammatory cytokine IL-10 and downregulation of multiple pro-inflammatory mediators, involving TNF-α, IFN-γ, MCP-1, IL-1β, IL-6, IL-8, IL-12, IL-12p70, and IL-17 A.

### Suppression of oxidative stress

Ulcerative colitis is characterized by a pathological imbalance between excessive reactive oxygen species (ROS) production and impaired antioxidant defenses [122]. Excessive ROS, including superoxide anions, hydrogen peroxide, and hydroxyl radicals, induce lipid peroxidation, protein oxidation, and DNA damage, exacerbating mucosal injury and disrupting epithelial barrier integrity [123]. This damage increases intestinal permeability, facilitating bacterial translocation and further immune activation. Concurrently, colitis patients display impaired antioxidant defenses, with reduced levels of glutathione, superoxide dismutase, and catalase, diminishing their ability to neutralize ROS and exacerbating oxidative damage [124]. Furthermore, ROS activate key pro-inflammatory signaling pathways, amplifying the release of cytokines, thereby perpetuating a vicious cycle of inflammation and tissue injury [125]. Oxidative stress also interacts with other pathogenic mechanisms in colitis, including mitochondrial dysfunction and endoplasmic reticulum stress, compounding cellular damage and hindering mucosal repair [126]. Thus, oxidative stress is a critical driver of colitis pathogenesis, contributing to sustained inflammation, impaired healing, and progressive tissue damage, making it a key focus for future research and treatment development.

In the study from Zu et al., they extracted the natural exosome-like nanotherapeutics (NTs) in tea leaves by the combination of DC and SDGC, and probed the effect and mechanism of NTs against colitis [87]. The results showed that NTs alleviated colitis primarily through antioxidant effects. Mechanistically, NTs Mechanistically, NTs reduced oxidative stress by lowering MPO activity (a marker of neutrophil infiltration and ROS generation), decreasing ROS and MDA (a hallmark of lipid peroxidation) levels, and increasing the abundance of reduced GSH and the expression of HO-1(an antioxidant enzyme).

In the study of Eom et al., they isolated hemp root-derived nanovesicles (RNVs) by the DC method, and probed the role and mechanism of RNVs against colitis [90]. The experimental data showed that RNVs could treat colitis partly through reducing the level of NO and the expressions of oxidative stress markers including iNOS, COX-2 and 3-NT.

Yang et al. found that the anti-colitis action of GDNPs is partly attributed to the antioxidant mechanism [94]. GDNPs could lower oxidative stress via increasing SOD activity and MMP value and decreasing the levels of ROS and MDA. Further mechanism study indicated that GDNPs stimulate the p62–Nrf2–Keap1 pathway to mitigate LPS‑evoked oxidative injury through upregulating the expression of p-p62, Nrf2, GCLC, GCLM, HO-1, NQO1 and downregulating the expression of Keap1.

In the study of Choi et al., they extracted aloe-derived nanovesicles, comprising *aloe vera*-derived nanovesicles (VNVs), *aloe arborescens*-derived nanovesicles (ANVs) and *aloe saponaria*-derived nanovesicles (SNVs) by the DC method, and evaluated their effects and mechanisms in DSS-evoked colitis mice [102]. The results showed that the anti-colitis roles of VNVs, ANVs and SNVs are partly related to the modulation of oxidative stress. VNVs, ANVs and SNVs could exert anti-oxidant effects through decreasing the levels of oxidative stress markers NO, COX-2 and 3NT.

Among the research from Tinnirello et al., they isolated industrially produced lemon nanovesicles (iLNVs) by the joint use of UC and UF, and investigated the potential mechanism of iLNVs against colitis [105]. The experimental proofs manifested that iLNVs could activate antioxidant responses to exert anti-colitis effect via reducing MPO activity and increasing the level of Nrf2.

During the experiment of Gao et al., they obtained *Momordica charantia*-derived extracellular vesicles (MCEVs) by the joint of DC and SDGC, and demonstrated that MCEVs exert therapeutic effects on colitis by mitigating oxidative stress [108]. In vitro experiments reveal that MCEVs effectively scavenge ROS, reducing oxidative damage in macrophages exposed to H₂O₂. Transcriptomic analysis further indicates that MCEVs modulate mitochondrial function by upregulating anti-apoptotic proteins (e.g., Bcl-2) and downregulating pro-apoptotic proteins (e.g., Bax), thereby preserving mitochondrial integrity and preventing ROS-induced cell death. Additionally, MCEVs contain bioactive compounds such as β-carotene and phosphatidylcholine, which contribute to their antioxidant capacity. In vivo, orally administered MCEVs accumulate in inflamed colonic tissues, significantly lowering ROS levels and alleviating DSS-induced colitis symptoms. These findings highlight MCEVs as a promising natural nanotherapeutic strategy for colitis treatment, primarily through their potent antioxidative properties.

Among the research of Li et al., they separated *Folium Artemisiae Argyi*-derived exosome-like nanovesicles (FAELNs) by the integration of UC and SEC, and further found that FAELNs could significantly mitigate colitis by suppressing oxidative stress [109]. In vitro, FAELNs scavenge ROS in LPS-stimulated macrophages and intestinal cells, reducing oxidative damage. They modulate apoptosis-related proteins (increasing Bcl-2 and decreasing Bax), indicating protection against ROS-induced cell death. In vivo, FAELNs decrease MPO activity (a neutrophil-derived oxidative stress marker) in DSS-induced colitis mice. In addition, their antioxidant effects are attributed to bioactive components like flavonoids and phosphatidylcholine.

### Adjustment of immune response

The immune response plays a central role in colitis development, characterized by dysregulated macrophage polarization and T cell differentiation [127]. Macrophages demonstrate a distinct polarization shift toward the pro-inflammatory M1 phenotype in active colitis, marked by increased production of IL-12, IL-23, and TNF-α, while anti-inflammatory M2 macrophages expressing IL-10 and TGF-β are significantly reduced [128]. This imbalance is accompanied by pathogenic T cell responses, with excessive Th1 and Th17 cell activation driving inflammation through IFN-γ and IL-17 A secretion, respectively [129]. Notably, regulatory T cells (Tregs) exhibit functional impairment in their immunosuppressive capacity, failing to control the exaggerated immune activation. The crosstalk between polarized macrophages and T cells creates a self-amplifying inflammatory loop. This immune axis disruption is particularly evident in the colonic mucosa, where infiltrating macrophages and T cells form characteristic inflammatory infiltrates [130]. Therapeutic strategies targeting these pathways, including anti-IL-12/23 biologics and Treg-promoting approaches, demonstrate the critical importance of immune rebalancing in colitis treatment [131].

**Influence on macrophages** TNVs from turmeric were shown by Gao et al. to significantly modulate macrophage polarization in colitis treatment [92]. In vitro experimental results manifested that M2 macrophages’ typical indicator CD206^+^ rate markedly elevated following TNVs in LPS- and IFN-γ-stimulated F4/80^+^CD11b^+^ macrophages. Furthermore, TNVs improved the positive rate of CD206. In vivo experimental results revealed that TNVs intervention dramatically lowered the population of activated F4/80^+^ CD11b^+^ macrophage from colonal lamina propria of colitis mice. Moreover, TNVs reduced the positive rate of M1 macrophages’ typical indicator CD16/32, and elevated the positive rate of CD206, gated on F4/80^+^ CD11b^+^ macrophages. Altogether, TNVs could restrain macrophage polarisation into M1 phenotypes, thus modulating enteric mucosa immunity.

The ginseng-derived nanoparticles GEN, as investigated by Kim et al., demonstrated potent effects on macrophage polarization during colitis treatment. investigated the change of macrophage polarization during the period of GEN treating colitis [93]. The results showed that GEN prominently downregulated IL-6 in the M1-polarised RAW264.7 cells in a concentration-reliant mode. Moreover, the M2 macrophage indicator CD206 was markedly upregulated following treatment of the cells with GEN.

Yang et al. demonstrated the important role of macrophage polarization in the process of GDNPs against colitis [95]. In vitro experimental results showed that GDNPs influenced the polarization of RAW264.7 macrophages by reducing the level of M1 polarization marker CD86 and increasing the level of M2 polarization marker CD206. Additionally, the expression of polarization-related CD206 and TGF-β were prominently increased, and the expression of polarization-related CD86 and iNOS were markedly decreased. In vivo experimental results displayed that GDNPs dramatically lowered CD86 expression and increased CD206 expression in the colonal mucosa and lamina propria on colitis mice. Furthermore, GDNPs significantly reduced the M1/M2 macrophage ratio, thus decreasing enteral inflammation in colitis murines.

Li et al. uncovered marked changes in the distribution of immune cells after HELNs treatment in comparison with the DSS-elicited colitis mice [104]. In particular, the entire population of macrophages (F4/80 + CD11b+) noticeably lowered, with a remarkable decline in the percentage of M1 macrophages (CD80+) and a rise in M2 macrophages (CD206+). This indicates that HELNs might restrain proinflammatory M1 macrophages development whilst encouraging anti-inflammatory M2 macrophages development. In addition, the percentages of dendritic cells (DCs, CD11c+) and neutrophils (CD11b + Gr-1+) were prominently lowered after HELNs administration. DCs exert a vital role for the presentation of antigens in the immune response process. Altogether, HELNs adjusts the immune system through diminishing the total quantity of macrophages, modulating macrophage polarisation, and lowering the quantity of DCs and neutrophils.

Contrasting with other findings, Lu et al. found that macrophage polarization exerts an important role in the treatment of colitis by PLDENs [106]. Administration of PLDENs dramatically enhanced the expression of M1 proinflammatory macrophage indicators involving TNF-α, IL-1β, IL-6, CD11c and CCL2. Meanwhile, PLDENs memorably upmodulated the levels of M2 proinflammatory macrophage indicators comprising CD206, IL-10, and YM1.

During the research of Zhu et al., they extracted *Andrographis paniculata*-derived exosome-like nanoparticles (APELNs) by the combined use of DC+SDGC, and explored its therapeutical efficacy and possible mechanism in alleviating DSS-elicited colitis [114]. It was found that APELNs alleviate colitis by modulating macrophage polarization. APELNs significantly reduce the pro-inflammatory M1 macrophage subset while promoting the anti-inflammatory M2 subset in the colon of DSS-induced colitis mice. Mechanistically, transcriptomic analysis reveals that APELNs upregulate IL-4R expression, which activates the PI3K-AKT and JAK-STAT signaling pathways. These pathways are critical for driving macrophage polarization toward the M2 phenotype, thereby suppressing inflammation. Flow cytometry and immunofluorescence confirm the shift from M1 to M2 macrophages, correlating with reduced pro-inflammatory cytokines (e.g., IL-12, TNF-α) and improved colitis symptoms. Thus, APELNs exert their anti-colitis effects by rebalancing M1/M2 macrophage polarization through IL-4R-mediated signaling.

**Other immune cells** Beyond macrophage polarization, DELNs also modulate other immune cell populations. In the study of Deng et al., they prepared broccoli-derived nanoparticles (BDNs) by the conjunction of DC, SEC and SDGC, and explored the action and mechanism of BDNs on colitis [86]. It was found that BDNs exert potent anti-inflammatory effects in colitis by modulating dendritic cell (DC) function through AMP-activated protein kinase (AMPK) activation. Oral administration of BDNs significantly attenuated disease severity in both DSS-induced and T cell transfer colitis models, as evidenced by reduced weight loss, colon shortening, and histological damage. Mechanistically, BDNs were preferentially taken up by intestinal DCs, where they activated AMPK signaling while inhibiting mTOR/S6K pathway, thereby inducing a tolerogenic DC phenotype characterized by upregulated PD-L1/PD-L2 and downregulated CD80/CD86/CD40 expression. This DC reprogramming led to decreased production of pro-inflammatory cytokines (TNF-α, IFN-γ, IL-17 A, IL-12, IL-23) and increased anti-inflammatory mediators (IL-10, TGF-β). Furthermore, BDNs inhibited monocyte recruitment by reducing CCL2/CCL20/CXCL1 chemokines and prevented Gr1 + monocyte differentiation into inflammatory CD11b + DCs. Crucially, the protective effects were AMPK-dependent and mediated by sulforaphane (SFN), a key bioactive component of BDNs, as SFN-depleted BDNs or AMPK-deficient DCs failed to confer protection. These findings demonstrate that BDNs represent a novel dietary-derived therapeutic strategy for colitis by promoting DC-mediated immune tolerance through AMPK activation.

Based on the investigation of Zhu et al., they obtained *Portulaca oleracea*-derived exosome-like nanoparticles (PELNs) by the joint of DC and SDGC, and probed the role and mechanism of PELNs towards colitis [99]. The results showed that PELNs could alleviate colitis partly through down-regulating the expression of Zbtb7b in routine CD4^+^ T cells, causing the transformation of routine CD4^+^ T cells into double-positive CD4^+^CD8^+^ T cells.

During the research of Kim et al., they obtained *Boehmeria japonica*-derived exosome-like nanoparticles (BJ-NPs) by the triple integration of DC, UF and SDGC, and explored the action and mechanism of BJ-NPs on colitis [107]. The study demonstrated that BJ-NPs exhibit remarkable immunomodulatory properties by significantly elevating IL-10 expression, which promotes the generation of DCs characterized by downregulated maturation markers (CD80/CD86/MHC-II). The modified DCs critically influenced T-cell responses, with flow cytometry data demonstrating that BJ-NPs treatment significantly decreased pro-inflammatory Th1 (CD3 + CD4+IFN-γ+) and Th17 (CD3 + CD4+IL-17 A+) cell numbers while increasing immunosuppressive CD3 + CD4+Foxp3 + regulatory T cells (Tregs).

In the study of Yang et al., they prepared *Coptis chinensis*-derived extracellular vesicle-like nanoparticles (Cc-ELNs) by the integrated approach of DC, UF, and UC, and probed the role and mechanism of Cc-ELNs on colitis [112]. The results showed that Cc-ELNs could prominently alleviate colitis via lowering neutrophil recruitment and restraining neutrophil extracellular traps (NETs) establishment. Moreover, through repressing NET formation, Cc-ELNs attenuated pyroptosis in enteric epithelia cells (IECs) and boosted the proliferation of both IECs and enteric stem cells (ISCs).

### Improvement of gut barrier function

The intestinal epithelial barrier, composed of tightly interconnected epithelial cells and overlying mucus layer, plays a fundamental role in colitis development [132]. Key structural alterations include: (1) disruption of tight junction (TJ) proteins, with characteristic Claudin-2 upregulation increasing pore pathway permeability and Occludin downregulation impairing barrier selectivity [133]; (2) significant reduction in goblet cell numbers and associated mucus layer thinning, particularly the inner sterile mucus layer composed of mucins [134]; (3) abnormal distribution and reorganization of TJ strands observed by electron microscopy [135]. These structural defects lead to increased intestinal permeability, as demonstrated by elevated lactulose/mannitol ratios in colitis patients. The compromised barrier allows luminal antigen penetration, initiating and perpetuating mucosal inflammation [136]. Notably, the spatial organization of TJ proteins is disrupted, with aberrant cytoplasmic internalization of ZO-1 and Occludin in inflamed mucosa. Goblet cell depletion not only reduces mucus production but also alters mucin glycosylation patterns, further impairing barrier function [137]. These morphological and functional changes in the intestinal barrier represent critical therapeutic targets for colitis management.

Eom et al. found that the defensive effect of hemp root-derived nanovesicles RNVs on colitis is tightly connected with the modulation of TJ proteins (e.g., ZO-1, Claudin-4 and Occludin) and adherent junction (AJ) proteins (e.g., E-cadherin and α-tubulin) in the intestine [90].

Yang et al. demonstrated that GDNPs mitigate colitis partly through defending the intestinal epithelia barrier [95]. Specifically, GDNPs reduced enteric penetrability and improved enteric mucosa injury. GDNPs hindered the diffusion of DAO from the enteric mucosa into the bloodstream, thereby mitigating injury to the enteric mucosa. GDNPs also prevented the extravasation of 5-HT and SP from the enteric mucosa into the bloodstream during gut stimulation and lowered the sensitivity of the enteric mucosa. Moreover, GDNPs markedly increased TJ proteins (e.g., ZO-1 and Occludin) in enteral‌ tissues.

According to the experiment of Wang et al., they prepared garlic-derived exosome-like nanoparticles (GELNs) by the combination of UC and SDGC, and inspected the action and mechanism of GELNs on colitis [98]. The proofs displayed that the efficacy of GELNs towards colitis is partly related to the suppression of colonic barrier impairment through increasing the levels of TJ proteins (e.g., ZO-1 and Occludin) and Mucin-2 protein.

Han et al. proved that the curative effect of NPs towards colitis is closely linked to the mucosal cell-cell junction [100]. In particular, NPs prominently increased the DSS-evoked decline of TJ proteins (e.g., ZO-1 and Claudin-1) and AJ protein E-cadherin, and decreased the expression of p-STAT3.

In the study from Choi et al., they demonstrated that the anti-colitic roles of VNVs, ANVs and SNVs are prominently connected with the restoration of epithelial barrier function through up-modulating the levels of TJ proteins (e.g., ZO-1, Claudin-4 and Occludin) and AJ proteins (e.g., γ-catenin, α-tubulin and E-cadherin) to downgrade intestinal epithelia permeability [102].

Zhu et al. proved that APELNs prominently suppresses colitis partly through reducing intestinal penetrability and preserving intestinal barrier integrality via up-regulating TJ proteins (e.g., ZO-1, Claudin-1 and Occludin) and Mucin-2 protein [114].

Furthermore, multiple studies have documented that TNVs from turmeric [92], GENs from garlic [97], HELNs from *Houttuynia cordata* [104], PLDENs from *Pueraria lobata* [106], MCEVs from *Momordica charantia* [108], FAELNs from *Folium Artemisiae Argyi* [109], AMEVLPs from *Atractylodes macrocephala* [110], CELNs and RELNs from *Zanthoxylum bungeanum* [111] all significantly enhance the expression of key junctional proteins including ZO-1, Occludin, Claudin-1, and Mucin-2 in colonic epithelial cells. This collective action reinforces the intestinal barrier by strengthening intercellular connections through TJ proteins (ZO-1, Occludin, Claudins), maintaining epithelial adhesion via AJ components, and preserving the mucus layer through Mucin-2 and E-cadherin upregulation. The consistent findings across these studies suggest a conserved mechanism whereby plant nanovesicles target junctional complexes to reduce intestinal permeability, prevent bacterial translocation, and ultimately mitigate colitis progression.

### Reshaping intestinal flora

The gut microbiota plays a pivotal role in ulcerative colitis pathogenesis through complex host-microbe interactions [138]. Colitis patients exhibit characteristic dysbiosis, marked by reduced microbial diversity, Depletion of beneficial commensals (e.g., SCFA-producing *achnospiraceae*, *Bifidobacterium* and *Akkermansia*), and expansion of pro-inflammatory Proteobacteria (e.g., adherent-invasive *Escherichia coli*) [139, 140]. This imbalance disrupts mucosal homeostasis through multiple mechanisms: (1) diminished production of anti-inflammatory short-chain fatty acids (SCFAs, particularly butyrate) due to loss of commensal Clostridia species [141]; (2) increased mucosal adherence and invasion by pathobionts, triggering TLR/NF-κB-mediated inflammation [142]; and (3) altered bile acid metabolism affecting farnesoid X receptor signaling [143]. These findings highlight microbial modulation as a promising therapeutic strategy for restoring immune homeostasis in colitis.

Zu et al. found that the anti-colitis action of NTs in tea leaves is closely related to the regulation of gut flora [87]. Specifically, NTs could enhance the abundances of probiotics involving Lachnospiraceae, *Bifidobacterium* and *Akkermansia*, and reduce the Firmicutes/Bacteroidetes ratio and the abundances of Proteobacteria, *Oscillibacter*, *Helicobacter*, *Mucispirillum*, *Brachyspira*, *Desulfovibriom* and *Parasutterella* spp.

In the study by Sriwastva et al., MBELNs caused significant changes in gut microbiota [89]. Specifically, MBELNs elevated the abundances of Dehalobacteriaceae, Lachnospiraceae, Porphyromonadaceae, Prevotellaceae, Pseudomonadaceae, Verrucomicrobiaceae, etc., and lowered the abundances of Bacteroidaceae, Odoribacteraceae, Lactobacillaceae, Turicibacteraceae, Alcaligenaceae, Helicobacteraceae, Mycoplasmataceae, and *Corynebacterium*, etc. Moreover, MBELNs possessed species-specific growth inhibitory roles towards virulent *Listeria (L.) monocytogenes-EGD* (*Lis-EGD*).

Gao et al. proved that TNVs could modulate the gut microflora to exert excellent therapeutical effect on colitis through increasing the amounts of *Akkermansia*, *Lactobacillus*, *Bifidobacterium* and Clostridia_UCG-014, and decreasing the amounts of *Bacteroides*, *Escherichia-Shigella*, *Helicobacter* and *Staphylococcus* [92].

Kim et al. proved that the therapeutic effect of GEN on colitis is partly related to the adjustment of intestinal flora including elevating the abundances of *Lactobacillus* and *Alistipes*, and lowering the Firmicutes/Bacteroidota ratio and the abundances of *Helicobacter*, *Oscillibacter* and *Ruminococcus* [93].

Based on the research of Zhu et al., they obtained garlic-derived exosome-like nanovesicles (GENs) by the integration of SDGC and UC, and explored the function and mechanism of GENs against colitis [97]. The experimental results manifested that GENs could affect gut microbiota to treat colitis through up-regulating the levels of Firmicutes, Lachnospiraceae and gut microbiota-generated SCFAs (e.g., acetic acid, butyric acid, propionic acid, valeric acid, isobutyric acid and isovaleric acid), and down-regulating the levels of Proteobacteria, *Helicobacter*, *Escherichia-Shigella* and *Akkermansia*.

Wang et al. proved that the efficacy of GELNs towards colitis is closely related to the adjustment of intestinal microbiome. In particular, GELNs elevates the quantities of *Eubacterium ruminantium*, *Insolitispirillum*, *Megasphaera*, *Acetatifactor*, *Bacteroides* and especially *Bacteroides thetaiotaomicron*, and lowers the numbers of *Anaerostipes*, *Anaeroplasma*, *Ileibacterium* and *Dubosiella* [98].

Zhu et al. proved that the anti-colitis effect of PELNs is significantly related to the modulation of intestinal microbiota [99]. Specifically, at the phylum level, Firmicutes and Patescibacteria exhibited increased abundances and Proteobacteria and Deferribacteres exhibited decreased abundances after PELNs administration. At the family level, PELNs elevated the levels of Lachnospiraceae, Ruminococcaceae, Muribaculaceae, etc., and lowered the levels of Enterobacteriaceae, Deferribacteraceae, Enterococcaceae, etc. At the genus level, PELNs enhanced the amounts of *Lactobacillus*, Lachnospiraceae_NK4A136_group, Ruminococcaceae_UCG-014, etc., and the amounts of *Escherichia-Shigella*, *Mucispirillum*, *Enterococcus*, etc. At the species level, PELNs improved the numbers of *Lactobacillus_gasseri*, *Lactobacillus_reuteri*, *Alistipes_inops*, etc., and depressed the numbers of *Bacteroides_thetaiotaomicron*, *Mucispirillum_*sp*_*69, *Enterococcus_faecalis*, etc.

Among the study of Kang et al., they isolated *Allium tuberosum*-derived exosome-like nanovesicles (ADNs) by the integration of UF and PBP, and probed the role and mechanism of ADNs towards colitis [103]. The results showed that the action of ADNs against colitis is tightly connected with the modulation of gut microbiota and its product SCFAs. Concretely, ADNs increases the levels of Bacteroidaceae, Lactobacillaceae, *Lactobacillus*, *Limosilactobacillus* and SCFAs (e.g., acetic acids, butyric acids), and decreases the Firmicutes/Bacteroidota ratio and the levels of Proteobacteria and *Clostridia*_UCG.

Li et al. found that the inhibitory effect of HELNs on colitis is partly related to the regulation of gut flora [104]. Specifically, HELNs medication elevates intestinal microbiome diversity, adjusts the composition of DSS-evoked enteric microflora in murines, diminishes harmful bacteria (e.g., *Candidatus_Saccharimonas*, *Escherichia-Shigella*, *Staphylococcus* and *Helicobacter*), and enhances the abundances of beneficial bacteria (e.g., Oscillospiraceae, *Lactobacillus*, *Odoribacter*, *Enterorhabdus*, *Solibacillus*, *Lactobacillus murinus* and *Lactobacillus apodemi*) to acquire a novel balance in the enteric ecosystem.

Tinnirello et al. demonstrated that the function of iNVs on colitis is partly related to the adjustment of gut microflora, involving the up-regulation of Lachnospiraceae NK4B4 and the down-regulation of *Enteractinococcus* and *Acetatifactor* [105].

According to the investigation of Lu et al., they acquired *Pueraria lobata*-derived exosome-like nanovesicles (PLDENs) by the conjunction of DC and UF, and inspected the action and possible mechanism of PLDENs on colitis [106]. The results proved the therapeutic effect of PLDENs towards colitis. This efficacy is partly associated with the modulation of gut microbiota through raising the levels of norank_f_Muribaculaceae, Muribaculaceae, Lactobacillaceae, Lachnospiraceae, Akkermansiaceae, *Lactobacillus*, etc., and lowering the quantities of Proteobacteria, Verrucomicrobiota, Rikenellaceae, Enterobacteriaceae, Helicobacteraceae, *Escherichia-Shigella*, etc.

During the experiment of Gao et al., they obtained *Momordica charantia*-derived extracellular vesicles (MCEVs) by the joint of DC and SDGC, and investigated the effect and mechanism of MCEVs against colitis [108]. The evidences manifested that MCEVs possess great potential as a candidate drug for colitis remedy. This therapeutic effect is partly linked to the regulation of intestinal microflora including increasing the levels of *Muribaculum* and *Alistipes* and decreasing the Firmicutes/Bacteroidota ratio and the amount of *Escherichia-Shigella*, and the adjustment of indole-related metabolites of gut flora.

Among the research of Li et al., they separated *Folium Artemisiae Argyi*-derived exosome-like nanovesicles (FAELNs) by the integration of UC and SEC, and explored the role and mechanism of FAELNs towards colitis [109]. The proofs displayed that FAELNs could efficiently alleviate DSS-induced colitis mice. This function is partly related with rectifying the unbalance of gut microflora, including up-regulating the levels of Lachnospiraceae, *Roseburia*, *Oscillibacter* and down-regulating the ratio of Firmicutes/Bacteroidota and the amounts of Proteobacteria, Prevotellaceae and *Erysipelatoclostridium*.

In the study of Tan et al., they prepared *Atractylodes macrocephala*-derived extracellular vesicles-like particles (AMEVLPs) by the combination of DC and UF, and probed the effect and mechanism of AMEVLPs on colitis [110]. The results showed that AMEVLPs could efficiently adjust the intestinal flora, thus prominently boosting the therapeutic effect on colitis. This is acquired via elevating the alpha diversity of intestinal flora, restoring beneficial species (e.g., Firmicutes, *Bacteroides* and *Alistipes*), and suppressing the harmful species (e.g., Proteobacteria and *Escherichia-Shigella*), which in turn modify tryptophan metabolism, resulting in a rise of indole derivatives (e.g., L-Tyrosine, Cinchophen, and 1-Methyladenosine) in the colons.

Zhu et al. found that the amelioration of APELNs on colitis is partly associated with intestinal microflora homeostasis [114]. In details, APELNs markedly improved the Lactobacillaceae/Staphylococcaceae ratio and the contents of Firmicutes, Lactobacillaceae, *Staphylococcus*, *Lactobacillus* and *Lactobacillus murinus*, and lowered the level of *Aeromonas veronii* B565.

Based on the study of Tuo et al., they prepared tangerine peel exosome-like nanoparticles (TPELNs) by the combination of DC and PBP, and inspected the action and mechanism of TPELNs towards colitis [115]. The results displayed that the anti-colitis role of TPELNs is notably linked to the modulation of gut flora. Specifically, TPELNs intervention increased the number of OTUs, restored microbial diversity and richness, regulated the Firmicutes/Bacteroidota ratio to near normal levels, increased the proportion of beneficial bacteria such as *Lactobacillus*, *Bifidobacteria* and *Bifidobacterium*, reduced the proportion of harmful bacteria such as *Escherichia−Shigella*, and upregulated the abundance of bacterial genera such as norank_o_Gastranaerophilales and UCG-009.

### Activation of autophagy

Autophagy plays a dual role in ulcerative colitis, serving as both a protective mechanism and potential therapeutic target [144]. Under physiological conditions, this cellular recycling process maintains intestinal homeostasis by clearing damaged organelles, regulating ER stress, and controlling bacterial clearance [145]. However, genetic defects (e.g., ATG16L1 mutations) and environmental triggers disrupt autophagic flux in colitis, leading to pathological consequences [146]. Impaired autophagy in epithelial cells compromises barrier function through defective tight junction turnover, while in immune cells it promotes inflammasome activation and excessive IL-1β/IL-18 production [147]. These alterations create a vicious cycle of sustained inflammation and impaired mucosal repair [148]. Therapeutic strategies targeting autophagy regulation show promise for breaking this pathogenic cycle in colitis treatment.

Among the experiment of Yang et al., GDNPs have been demonstrated to significantly activate autophagy [95]. Through comprehensive in vitro and in vivo studies, GDNPs were shown to upregulate key autophagy markers including LC3-II, Beclin-1, and Atg7 in RAW264.7 macrophages, indicating enhanced autophagic flux. Confocal microscopy analysis revealed a substantial increase in GFP-LC3 puncta formation in GDNPs-treated cells, while this effect was effectively suppressed by the autophagy inhibitor 3-methyladenine (3-MA), confirming the specific induction of autophagy by GDNPs. The mechanism underlying GDNPs-mediated autophagy activation involves modulation of the AKT/mTOR signaling pathway. GDNPs treatment led to significant downregulation of phosphorylated AKT and mTOR, key negative regulators of autophagy, thereby relieving their inhibitory effects on the autophagic process. Transmission electron microscopy further validated these findings by demonstrating increased formation of autophagic lysosomes in GDNPs-treated macrophages.

### **Safety of PDELNs in the treatment of colitis**

Compared with liposome nanoparticles and mammalian extracellular vesicles (MEVs), PDELNs exhibit superior biocompatibility, safety, and lower immunogenicity [149]. These natural nanoparticles are derived from edible plants commonly consumed in human diets, with many source plants already approved as food additives [150]. Structurally, PDELNs share key characteristics with MEVs, including a typical size range of 30–400 nm, negatively charged surface properties, and a phospholipid bilayer structure that facilitates efficient cellular uptake and biological barrier penetration [151]. Importantly, PDELNs exhibit an inability to cross the placental barrier, suggesting their potential as safer therapeutic options for pregnant patients [152].

At present, most of the researches on the effect and mechanism of PDELNs against colitis have conducted safety experiments, such as NTs, MBELNs, TNDPs, GDNPs and PELNs [153]. Safety investigation of these studies mainly involves cytotoxicity assay, measurement of body weights and colon lengths, organ coefficients inspection, H&E staining-based histological examination of main organs (e.g., heart, liver, spleen, lung and kidney), biochemical analyses (e.g., AST, ALT, BUN, CREA and UREA), etc. As systematically summarized in Table 3, the experimental evidence consistently confirms the safety and non-toxic nature of all currently characterized anti-colitis PDELNs, thereby substantiating their significant therapeutic potential for clinical translation.

**Table 3:** Safety of plant-derived exosome-like nanovesicles (PDELNs) against colitis

Names	Plant sources	Cells/Animals	Dosages	In vitro safety	In vivo safety (Treatment vs. Control)	References
NTs	Tea leaves	RAW264.7 cells CT-26 cells L929 cells FVB female mice	0.5, 1, 2, 4, 8 µg/mL 2 mg/kg per mouse	No cytotoxicity was found up to 8 µg/mL	a. Body weights, organ indices (heart, liver, spleen, lung, and kidney), spleen/body weight ratios showed no obvious variations; b. Blood parameters (lymphocyte, granulocyte and platelet numbers) were all in the normal ranges; c. H&E-stained sections of GIT tissues (stomach, duodenum, jejunum, ileum, cecum, and colon) and five main organs (heart, liver, spleen, lung, and kidney) displayed no obvious abnormalities; d. Liver function’s indexes (AKP and AST) and kidney function’s indexes (UA, CREA and BUN) exhibited no significant changes.	[87]
DONPs	*Dendrobium officinale*	RAW264.7 cells FVB female mice	2.5, 5, 10, 20 µg/mL 0.5, 1 mg/kg	No cytotoxicity was found up to 20 µg/mL	a. Body weights and organic coefficients (heart, liver, spleen, lung, and kidney) showed no obvious variations; b. Blood parameters (leukocyte, lymphocyte, monocyte and granulocyte numbers) were all in the normal ranges; c. H&E-stained sections of GIT tissues (duodenum, jejunum, ileum, cecum, and colon) and five main organs (heart, liver, spleen, lung, and kidney) displayed no obvious abnormalities; d. Liver function’s indexes (ALT and AST) and kidney function’s indexes (CREA and BUN) showed no significant changes.	[88]
MBELNs	Mulberry bark	C57BL/6J mice	2 × 10^9^, 1 × 10^10^ particles / 100 µl/dose/mouse/d	/	a. Body weights, skin rash and fecal discharge presented no significant differences; b. Morphology of internal organs and gut tissue microscopic structure displayed no obvious abnormalities; c. Blood parameters (cholesterol and triglycerides) and liver function’s indexes (ALT and AST) showed no significant changes.	[89]
TDNPs	Turmeric	Raw 264.7 cells colon-26 cell FVB/NJ mice	1, 10, 20, 50, 100 µg/mL 3 mg/dose	No cytotoxicity was found up to 100 µg/mL	a. The weights of heart, liver, spleen, lung and kidney, and H&E-stained sections of the five main organs displayed no obvious abnormalities; b. Liver function’s indexes (ALT and AST) showed no significant changes.	[91]
TNVs	Turmeric	Raw264.7 cells HT-29 cells NCM 460 cells Zebrafish	3.12, 6.25, 12.5, 25, 50, 100 µg/mL 100, 200, 300, 400, 500 µg/mL	No cytotoxicity was found up to 100 µg/mL	a. Organ coefficients of heart, liver, lung and kidney, and H&E-stained sections of five main organs (heart, liver, spleen, lung and kidney) displayed no obvious abnormalities; b. Liver function’s indexes (ALT and AST) and kidney function’s indexes (CREA and BUN) showed no significant changes; c. Zebrafish survival has not been affected up to 500 µg/mL of TNVs.	[92]
GEN	*Panax ginseng*	Balb/C male mice	1 mg per mouse	/	a. Liver function’s indexes (ALT and AST) showed no significant changes.	[93]
GDNPs	*Panax ginseng*	C57BL/6J male mice	2 mg per mouse	/	a. Body weights, and organ coefficients of heart, liver, spleen, lung and kidney manifested no significant differences; b. H&E-stained sections of five main organs (heart, liver, spleen, lung and kidney) and GIT tissues (stomach, duodenum, jejunum, ileum, cecum and colon) displayed no obvious abnormalities; c. Liver function’s indexes (ALT, AST and TPII) and kidney function’s indexes (CREA and UREA) showed no significant changes.	[94]
PELNs	*Portulaca oleracea* L	C57BL/6 male mice	50, 100 mg/g	/	a. H&E-stained sections of five main organs (heart, liver, spleen, lung, and kidney) displayed no obvious abnormalities b. Liver function’s indexes (ALT and AST) and kidney function’s indexes (CREA and UREA) showed no significant changes.	[99]
VNVs ANVs SNVs	*Aloe vera* *Aloe arborescens* *Aloe saponaria*	T84 cells HT29 cells	1, 10, 50 µg/mL	No cytotoxicity was found up to 50 µg/mL	/	[102]
ADNs	*Allium tuberosum*	RAW264.7 cells	0.5, 1, 2, 5, 10 ng/mL	No cytotoxicity was found up to 10 ng/mL	/	[103]
HELNs	*Houttuynia cordata*	C57BL/6 male mice	5, 10 mg per mouse	/	a. The ratios of major organ (heart, liver, spleen, lung and kidney) weights to body weights manifested no marked differences; b. H&E-stained sections of five main organs (heart, liver, spleen, lung and kidney) displayed no obvious abnormalities; c. Liver function’s indexes (ALT and AST) and kidney function’s indexes (BUN and CREA) showed no significant changes.	[104]
BJ-NPs	*Boehmeria japonica*	Bone marrow-derived dendritic cells	10, 50, 100, and 200 µg/mL	No cytotoxicity was found up to 200 µg/mL	/	[107]
MCEVs	*Momordica charantia*	RAW264.7 cells FHC cells C57BL/6 male mice	0.5, 1, 2.5, 5, and 10 µg/mL 30 mg/kg	No cytotoxicity was found up to 10 µg/mL	a. Body weights, and H&E-stained sections of five main organs (heart, liver, spleen, lung and kidney) manifested no obvious abnormalities; b. Liver function’s indexes (ALT, AST, ALB, GLO and TP) and kidney function’s index UREA showed no significant changes; c. Blood indices (leukocyte, lymphocyte, neutrophil, erythrocyte, hemoglobin, platelet) were all in the normal ranges.	[108]
FAELNs	*Folium Artemisiae Argyi*	RAW264.7 cells HT-29 cells NCM 460 cells Balb/c female mice	1, 10, 20, 50, 100 µg/mL 25, 50 mg/kg	No cytotoxicity was found up to 100 µg/mL	a. The ratios of organ (heart, liver, spleen, lung and kidney) weights to body weights manifested no marked differences; b. H&E-stained sections of five main organs (heart, liver, spleen, lung and kidney) displayed no obvious abnormalities c. Liver function’s indexes (ALT and AST) and kidney function’s indexes (BUN and CREA) showed no significant changes.	[109]
AMEVLP	*Atractylodes macrocephala*	RAW264.7 cells C57BL/6J female mice	1, 2, 5, 10, 15, 20 µg/mL 0.5, 2 mg/kg/d	No cytotoxicity was found up to 20 µg/mL	a. Body weights, and H&E-stained sections of five main organs (heart, liver, spleen, lung and kidney) manifested no obvious abnormalities; b. Liver function’s indexes (ALT and AST) and kidney function’s indexes (UREA and CREA) displayed no significant changes; c. Blood parameters (leukocyte, erythrocyte, PLT, hemoglobin, neutrophil, lymphocyte numbers) were all in the normal ranges.	[110]
CELNs RELNs	*Zanthoxylum bungeanum*	C57BL/6J male mice	1 × 10^11^ particles/kg/d	/	a. H&E-stained sections of five main organs (heart, liver, spleen, lung and kidney) showed no obvious abnormalities; b. Liver function’s indexes (ALT and AST), kidney function’s indexes (CREA and UREA), and heart function’s indexes (CK, CK-MB, LDH and α-HBDH) displayed no significant changes.	[111]
Cc-ELNs	*Coptis chinensis*	C57BL/6J male mice	50 µg per mouse	/	a. Body weights, and organ indices (heart, liver, spleen, lung, kidney and brain) manifested no significant differences; b. H&E-stained sections of GIT tissues (duodenum, jejunum, ileum, cecum, rectum and colon) and seven main organs (heart, liver, spleen, lung, kidney, brain and bone marrow) showed no obvious abnormalities; c. Liver function’s indexes (ALT, AST and TP) and kidney function’s indexes (CREA and UREA) displayed no significant changes.	[112]
PM-EVLPs	*Prunus mume*	C57BL/6 female mice	0.5, 1.5 × 10^10^ particles/mL	/	a. Body weights, colon lengths, and H&E-stained sections of six main organs (heart, liver, spleen, lung, kidney and colon) displayed no obvious abnormalities b. Liver function’s indexes (ALT and AST) and kidney function’s indexes (CREA and UREA) showed no significant changes.	[113]
APELNs	*Andrographis paniculata*	NCM460 cells HCT116 cells C57BL/6 mice	0.5, 2.5 mg/mL 0.5 mg per mouse	No cytotoxicity was found up to 24 mg/mL	a. H&E-stained sections of five main organs (heart, liver, spleen, lung and kidney) showed no obvious abnormalities; b. Erythrocyte count, heart function’s index CK-MB, liver function’s indexes (ALT and AST), and kidney function’s indexes (CREA and UREA) displayed no significant changes	[114]

### Conclusion and perspective

The current research highlights the multifaceted therapeutic potential of PDELNs in the treatment of colitis, with their efficacy primarily driven by a spectrum of interrelated biological mechanisms. Among these, anti-inflammatory action, gut microbiota remodeling, and immune response modulation emerge as the core mechanisms underlying their protective effects. Specifically, PDELNs consistently demonstrate the ability to suppress pro-inflammatory cytokine production (e.g., TNF-α, IL-6, IL-1β), upregulate anti-inflammatory mediators (e.g., IL-10), inhibit key inflammatory signaling pathways (e.g., NF-κB, NLRP3, MAPK), and restore the balance between pro-inflammatory (M1) and anti-inflammatory (M2) macrophage polarization. Simultaneously, they contribute to the reconstruction of a healthy gut microbiota by increasing the abundance of beneficial microbes (e.g., *Akkermansia*, *Lactobacillus*, *Bifidobacterium*) and reducing pathogenic populations (e.g., *Proteobacteria*, *Helicobacter*), thereby enhancing mucosal homeostasis. Furthermore, PDELNs modulate immune dysregulation in colitis, characterized by excessive Th1/Th17 activation and impaired Treg function, through targeted effects on immune cell subsets, including macrophages, dendritic cells, and T lymphocytes.

Beyond these primary mechanisms, PDELNs also exert secondary but complementary effects​that collectively reinforce their therapeutic efficacy. These include improvement of intestinal barrier function (e.g., upregulation of TJ proteins like ZO-1 and Occludin, restoration of mucus layer integrity via Mucin-2), attenuation of oxidative stress (e.g., reduction of ROS, MDA, and iNOS; elevation of SOD, GSH, and Nrf2), and activation of autophagy (e.g., induction of LC3-II, Beclin-1, and autophagic flux, suppression of inflammasome hyperactivation). These secondary pathways address critical pathological features of colitis, such as mucosal permeability, oxidative damage, and defective cellular clearance, that synergize with core mechanisms to achieve comprehensive colitis alleviation.

A particularly promising aspect of PDELNs is their exceptional safety profile. Extensive preclinical evaluations have consistently demonstrated their advantages including biocompatibility, low immunogenicity, and absence of toxicity, that stem from their natural plant origins and structural similarity to mammalian extracellular vesicles. Unlike synthetic nanoparticles, PDELNs exhibit inherent biological compatibility while maintaining the ability to cross biological barriers, though importantly, they cannot penetrate the placental barrier, suggesting potential safety for use in pregnant patients.

Despite these advances, several critical challenges must be systematically addressed to facilitate the clinical translation of PDELNs. First, the primary limitation lies in the incomplete identification of the specific bioactive components within PDELNs that directly mediate their therapeutic effects. Although there are emerging reports implicating a few specific components, such as certain plant miRNAs and metabolites, these findings represent only a small fraction of PDELNs, and the active molecules in the vast majority of PDELNs remain uncharacterized. Therefore, a primary objective moving forward is to systematically characterize the key functional molecules (including lipids, nucleic acids, proteins, and metabolites) across various PDELN sources and to delineate their interactions with host immune or microbial targets. Such efforts are crucial not only for elucidating the precise mechanisms of action but also for enabling the rational design of optimized nanotherapeutics. Second, current preparation methods present a significant bottleneck. The gold-standard technique of differential and density gradient centrifugation is inefficient and difficult to scale, while all alternative methods suffer from distinct drawbacks regarding yield, purity, or cost. The development of integrated, scalable purification workflows and the establishment of rigorous, standardized quality control metrics are therefore critical priorities. Third, the long-term safety profile of PDELNs requires more rigorous investigation. Future toxicological studies must extend beyond conventional safety parameters to comprehensively assess chronic toxicity, biodistribution, and potential drug interactions using advanced models and technologies.

In conclusion, PDELNs represent a novel and promising class of nanotherapeutics for colitis, with their multi-targeted mechanisms and favorable safety profile positioning them as potential alternatives or adjuncts to existing treatments. Future directions should focus on engineering PDELNs for enhanced targeting specificity, particularly to inflamed colon tissues, and developing combinatorial approaches with conventional drugs. The multimodal mechanisms, natural origin, and demonstrated safety of PDELNs position them as a transformative therapeutic platform that bridges traditional phytotherapy and modern nanomedicine, offering new hope for colitis patients refractory to current treatments. As research progresses, we anticipate PDELNs will not only provide effective colitis management but may also serve as a model for developing plant-based nanotherapeutics for other inflammatory and autoimmune conditions, potentially revolutionizing our approach to chronic disease treatment.

## Data Availability

No datasets were generated or analysed during the current study.

## Abbreviations

AFM: Atomic force microscopy
AhR: Aryl hydrocarbon receptor
AKP: Alkaline phosphatase
ALB: Albumin
ALT: Alanine aminotransferase
AMPs: Antimicrobial peptides
AST: Aspartate aminotransferase
BCA: Bicinchoninic acid assay
BUN: Blood urea nitrogen
CD: Cluster of differentiation
CK: Creatine kinase
CK-MB: Creatine kinase-myocardial band
COX-2: Cyclooxygenase-2
CREA: Creatinine
cryo-EM: Cryo-electron microscopy
CUL1: Cullin 1
CYP1A1: Polypeptide 1
DC: Differential centrifugation
DGC: Density gradient centrifugation
eNOS: Endothelial nitric oxide synthase
ESCRT: Endosomal sorting complex required for transport‌
EXPO: Exocyst-positive organelle
GC-MS: Gas chromatography-mass spectrometry
GIT: Gastrointestinal tract
GLO: Globulin
GSH: Glutathione
α-HBDH: α-hydroxybutyrate dehydrogenase
H&E: Hematoxylin and eosin
HO-1: Hemeoxygenase-1
IC: Immunoaffinity capture
IDGC: Iodixanol density gradient centrifugation
IDO1: Indoleamine 2,3-dioxygenase 1
IECs: intestinal epithelial cells
IFN: Interferon
IL: Interleukin
IκB: Inhibitor of nuclear factor kappa B
ILVs: Intraluminal vesicles
iNOS: Inducible nitric oxide synthase
ISCs: Intestinal stem cells
LC-MS: Liquid chromatograph-mass spectrometer
LDH: Lactate dehydrogenase
LPS: Plasma endotoxin
MCP-1: Monocyte chemotactic protein 1
MDA: Malondialdehyde
MMP: Mitochondrial membrane potential
MPO: ‌Myeloperoxidase
MVB: Multivesicular body
NETs: Neutrophil extracellular traps
NF-κB: Nuclear factor kappa-B
Nrf2: Nuclear factor-erythroid 2-related factor 2
3NT: 3-nitrotyrosine
PBP: Polymer-based precipitation
PEG: Polyethylene glycol
PLT: Platelet
ROS: Reactive oxygen species
SDGC: Sucrose density gradient centrifugation
SEC: Size exclusion chromatography
SEM: Scanning electron microscopy
SOD: Superoxide dismutase
STAT3: Signal transducer and activator of transcription 3
TEER: Transepithelial electrical resistance
TEM: Transmission electron microscopy
TGF: Transforming growth factor
TNF-α: Tumor necrosis factor-alpha
UA: Uric acid
UC: Ultracentrifugation
UF: Ultrafiltration
UREA: Carbamide
ZO-1: Zonula occludens-1
